# Machine learning-based classification of roses using 18 SNP markers for optimized genebank management

**DOI:** 10.1186/s13007-025-01496-0

**Published:** 2026-01-06

**Authors:** Laurine Patzer, Marcus Linde, Thomas Debener

**Affiliations:** Institute of Plant Genetics, Section Molecular Plant Breeding, Leibniz University Hannover, Herrenhäuser Straße 2, 30419 Hannover, Germany

**Keywords:** Machine learning, SNP data, Rose, Classification, Clustering, PACE, Taxonomy, Supervised learning

## Abstract

**Background:**

Reliable classification of rose cultivars is complicated by their long and complex breeding history, frequent hybridization, and the coexistence of traditional horticultural categories with genetically heterogeneous groups. While molecular marker sets such as SSRs have been applied to assess genetic relationships, studies across cultivated roses and species are rare. Using 18 SNP markers on 1,345 accessions in combination with machine learning now offers an opportunity to systematically evaluate how well horticultural classes align with underlying genomic structure and to provide robust tools for the management of large germplasm collections.

**Results:**

Using a panel of SNP markers across 1,345 rose accessions from the Europa Rosarium Sangerhausen, multiple unsupervised (hierarchical, spectral, k-means, DBSCAN, HDBSCAN) and supervised (SVM, decision tree, naive Bayes, XGBoost) machine learning approaches were applied to identify genetic clusters and predict horticultural classifications. Across clustering methods, certain groups consistently emerged as genetically distinct, such as the alba and damask roses, which clustered together with low internal diversity, reflecting their shared historic origin. In contrast, tea, bengal, lutea, and remontant hybrids were repeatedly grouped together and predicted with high classification accuracies (up to 100%) but displayed high within-group diversity, which is consistent with complex breeding backgrounds. Miniature, kordesii, and rubiginosa hybrids also tended to cluster together, despite their differing horticultural labels. Overall, the labels obtained from unsupervised clustering were consistently confirmed by supervised models, which achieved balanced accuracies of up to 100%, highlighting the robustness of the observed groupings.

**Conclusions:**

Our results demonstrate that machine learning applied to SNP marker data can robustly resolve genetic relationships among rose cultivars and provide novel insights into the alignment of horticultural classifications with genomic structure. The high predictive accuracies obtained suggest that marker-based classification can serve as a reliable complementary tool for genebank management, cultivar identification, and reassessment of traditional rose categories.

**Supplementary Information:**

The online version contains supplementary material available at 10.1186/s13007-025-01496-0.

## Background

The genus *Rosa* belongs to the Rosaceae family and is best known for its ornamental and horticultural value. It represents a highly diverse group of approximately 200 species [1] and is widely distributed in Asia and, to a lesser extent, Europe, North America, and North Africa. This diversity is reflected in the wide range of flower colors, sizes, and growth habits, which are characteristics that have been extensively selected for use in ornamental breeding. Taxonomy within the genus *Rosa* is complex because of hybridization and polyploidy, making classification difficult. However, the classification system of Rehder (1949) [2], extended by Wissemann 1], is often used and divides the genus into four subgenera: *Hulthemia*, *Hesperrhodos*, *Platyrhodon*, and *Rosa* [1–3]. The subgenus *Rosa* contains 95% of all species and is divided into further sections such as *Pimpinellifoliae*, *Cinnamomeae*, *Caninae*, *Carolinae*, *Gallicanae*, *Synstylae*, *Indicae*, *Laevigatae*, *Bracteatae*, and *Banksiae* [2, 4]. It is assumed that only approximately 10 to 20 species, e.g., *R. alba*,* R. centifolia*,* R. damascena*,* R. gallica*,* R. moschata*,* R. pimpinellifloia*,* R. sempervirens*,* and R. wichuraiana*, have contributed to modern rose cultivars [5–7].

Domestication of roses probably started with the cultivation of rose species collected from the open field, allowing spontaneous hybridization between species from different parts of the world. Records of controlled crosses date back to the beginning of the nineteenth century [8]. A major turning point in breeding occurred with the introduction of the diploid *Rosa chinensis* to Europe in the late 18th century. Its hybridization with predominantly tetraploid European roses resulted in triploid tea hybrids that combined traits such as recurrent blooming, abiotic stress tolerance, double flowering, and novel colors [9]. The first hybrid tea rose variety, ‘La France’, was introduced by Guillot in 1867 and is considered to be the first modern rose. This era was followed by rapid diversification: polyantha roses emerged from crosses of tea roses with *R. multiflora* at the end of the 19th century, Floribunda roses from polyantha × tea hybrids, and miniature roses from the rediscovery of *R. roulettii* in 1920. The introduction of ‘Soleil d’Or’ in 1900 also enabled the incorporation of novel yellow hues derived from *Rosa foetida* [10].

As modern rose breeding progressed, traditional cultivar classes became increasingly intermingled. Breeders focused on specific horticultural traits and roses were grouped according to cultural and economic significance. Consequently, the distinction between groups began to blur, and existing classification schemes often failed to capture genetic diversity. Studies at the level of nuclear and chloroplast DNA [3, 11, 12] have shown that traditional taxonomic divisions reveal complexities that are not captured by morphological data alone. For instance, *Cinnamomeae*, *Carolinae*, and *Pimpinellifoliae* do not appear monophyletic, whereas *Indicae* and *Synstylae* are well supported by molecular data [3]. Similarly, Zhu et al. [13] reported that the traditionally defined sections *Chinenses (= Indicae)* and *Synstylae* are not monophyletic and show close phylogenetic relationships with the sections *Caninae* and *Gallicanae*. For genebanks tasked with preserving genetic diversity, this lack of genetically meaningful groupings presents a major challenge. With more than 34,000 granted plant variety rights in the ornamental crop sector within the EU [14], it is not feasible to conserve them all, and prioritization requires a clear understanding of genetic backgrounds. Identifying representative and diverse accessions efficiently is therefore essential for effective genebank management and long-term conservation.

Machine learning (ML) offers the possibility of identifying hidden patterns and nonlinear relationships in genetic data that are often overlooked by traditional taxonomic approaches, which typically rely on distance-based clustering (e.g., UPGMA, PCoA) or predefined classification criteria. Recent studies have demonstrated that machine learning can be successfully applied to classify plant genetic resources. Nasiri et al. [15] evaluated supervised and unsupervised algorithms in an SSR-based fingerprinting study of pea accessions and achieved high prediction accuracies, whereas Torabi-Giglou et al. [16] reported that ML approaches effectively classified potato populations and identified key markers underlying their genetic structure. These genetic data-driven approaches provide a better understanding of the genetic similarities and differences between roses, thereby supporting the prioritization and management of resources within genebanks to minimize the loss of genetic diversity.

The objective of this study was to explore which classification scheme most accurately reflected the underlying genetic structure of cultivated roses to support effective genebank management. Using data from Patzer et al. [17], which includes several thousand rose accessions genotyped with 18 highly informative SNP markers, we investigated the potential of unsupervised and supervised machine learning methods to (i) identify genetically coherent clusters between traditional cultivar groups and additional attributes such as breeder origin and release year, (ii) explore the predictability of these clusters, and (iii) investigate the genetic diversity within these clusters.

## Methods

### Plant material and genotyping

In this study, 1,345 rose samples from the Europa Rosarium Sangerhausen (ERS) were analyzed. The genotypic data utilized in our analysis originate from the work of Patzer et al. [17], in which 4,187 rose accessions were screened using 18 PCR Allele Competitive Extension (PACE) markers that were stringently selected for divergent chromosomal positions, high discriminating power and successful amplification across a wide range of genotypes. For our specific analysis, a subset of these accessions was selected on the basis of two criteria: (1) they originated exclusively from the ERS, and (2) they were successfully scored for all 18 PACE markers. Following these selection criteria, our final subset comprised 1,345 accessions. This targeted selection was designed to maximize the reliability and accuracy of our machine learning process while focusing on the ERS collection, where comprehensive information (breeder, horticultural group, year) about the accessions is available. The information on the accessions provided by the ERS consisted of a total of 45 different rose group labels and 151 different breeders and spanned the years from 1750 to 2016 (Additional File 1).

The detailed methods used for DNA isolation and the PACE assay have been described by Patzer et al. [17].

### Software

All analyses were conducted using R (version 4.4.2) [18] utilizing the packages listed in the following chapters. Graphical visualizations were created with the R packages `ggplot2´ [19], ‘patchwork’ [20], `RcolorBrewer´ [21], `cowplot´ [22], and `ggforce´ [23]. For data import and project organization, we used the packages `here´ [24] and `openxlsx´ [25]. Data wrangling and dataset organization were performed using `tidyverse´ [26].

### Identity-By-State (IBS) analysis

Genetic similarity among 1,345 rose accessions was assessed using Identity-By-State (IBS) based on 18 SNP markers per accession (allele dosage 0–4). Each accession was assigned to a horticultural class predefined by the ERS. SNP data were converted to an integer matrix, and sample identifiers were stored separately. The SNP matrix was then converted into a Genomic Data Structure (GDS) file using the R package SNPRelate [27] to facilitate efficient IBS computation. Pairwise IBS values between all accessions were calculated with the snpgdsIBS() function. To summarize genetic similarity at the class level, IBS values were averaged across all pairs of individuals between each pair of classes. Diagonal values of the resulting class-level IBS matrix were set to 1 to reflect complete identity within classes. The class-level IBS matrix was visualized as a heatmap with hierarchical clustering of both rows and columns using the pheatmap R package [28]. This approach highlighted patterns of genetic similarity and divergence among classes.

### Unsupervised learning and genetic clustering

Unsupervised learning methods were employed to identify patterns and substructures within the genetic data. Prior to clustering, the allele dosage values were standardized using z-transformation. A variety of clustering algorithms were applied to the scaled SNP matrix, including k-means clustering with k = 2 and 25 random starts for robustness and hierarchical clustering based on Ward’s method (ward.D2) and Euclidean distance with a predefined k = 4. Hierarchical clustering results were visualized using the ggdendro R package [29] to generate dendrograms for clear representation of cluster relationships. The number of clusters for k-means and hierarchical clustering was chosen based on prior knowledge about broad genetic subdivisions and dendrogram inspection (Additional File 2), and these choices were further supported by visual evaluation of PCA and UMAP projections to ensure that major clusters were clearly separated. Spectral clustering was performed using the specc() function from the R ‘kernlab’ package [30]. Here, the optimal number of clusters was determined using the average silhouette width (R package ‘cluster’ [31]). Density-based clustering carried out using DBSCAN and HDBSCAN (package ‘dbscan’ [32]) both using minPts = 8 and eps = 2.5 for DBSCAN. These parameters were selected to detect meaningful clusters while avoiding excessive merging of distinct genetic groups. Parameter tuning indicated that moderate changes to eps and minPts had little effect on the resulting cluster structures.

To assess the consistency among clustering methods, we generated upset plots using the R package ggupset [33] to visualise the degree of agreement and disagreement between methods.

Cluster assignments were projected onto both PCA and UMAP coordinates (calculated with the ‘factoextra’ and ‘umap’ packages [34, 35]) to facilitate interpretation of genetic group structures. To evaluate the biological relevance of the clusters, cluster memberships were compared to external categorical variables such as class, breeder, and year of release. Only groups containing at least five individuals were included in the comparative visualizations.

### Supervised learning

For supervised machine learning, the dataset was split into training and test data, with 80% of the data used for training and 20% for testing. To address class imbalance, the synthetic minority oversampling technique (SMOTE) implemented in the R package ‘caret’ [36] was applied to the training data. Four classification algorithms were trained and compared within the framework of the caret package [36]: support vector machine with radial basis function kernel (svmRadial) from the ‘e1071’ package [37], decision tree (‘rpart’ package [38]), naive Bayes (‘naivebayes’ package [39]), and extreme gradient boosting (xgbTree) from the ‘xgboost’ package [40] were employed to identify patterns and target variables. Model tuning was performed using 5-fold cross-validation within the training set, optimizing for overall accuracy. Performance metrics, including accuracy, Cohen’s kappa, balanced accuracy, and F1 score, were computed to assess model performance. Accuracy was defined as the proportion of correctly classified samples: $\:Accuracy=\:\frac{TP+TN}{TP+TN+FP+FN}$, where TP (true positives) and TN (true negatives) denote correctly classified samples of the positive and negative classes, respectively, and FP (false positives) and FN (false negatives) represent misclassified samples. Cohen’s kappa accounts for chance agreement was calculated as $\:k=\:\frac{{p}_{0}-{p}_{e}}{1-\:{p}_{e}}$, where $\:{p}_{0}$ is the observed agreement between the predicted and true labels and $\:{p}_{e}$ is the expected agreement by chance, derived from the marginal class probabilities. To address class imbalance, the balanced accuracy was calculated as the mean of the sensitivity and specificity: $\:Balanced\:accuracy=\frac{1}{2}(\frac{TP}{TP+FN}+\frac{TN}{TN+FP})$, and extended to the multiclass case by averaging the per-class recall values. The F1 score was computed as the harmonic mean of precision and recall with $\:F1=2*\frac{Precision*Recall}{Precision+Recall}$, where $\:Precision=\frac{TP}{TP+FP}$ and $\:Recall=\frac{TP}{TP+FN}$. The final model performance was assessed on the independent test set. Confusion matrices with both absolute and percentage-based values per reference class were generated and visualized, enabling detailed inspection of the classification errors and successes.

### Genetic diversity indices

SNP genotype data were stored in a dataframe, where each column represented one of the 18 SNP markers and each row contained the corresponding allele dosage (0–4) for a tetraploid individual. These data were converted into a genind object using the df2genind() function from the ‘adegenet’ R package [41, 42], which specifies codominant markers (type = “codom”) and tetraploidy (ploidy = 4). To enable population-level analyses and account for potential clonality, the genind object was subsequently converted into a genclone object using the as.genclone() function from the ‘poppr’ package [43]. Genetic diversity indices were calculated on the basis of SNP genotype data and cluster labels derived from unsupervised learning methods. For each accession, a cluster label was assigned if at least 75% of the accessions within a predefined rose group belonged to the same cluster. The following diversity metrics were computed using the poppr() function: number of multilocus genotypes (MLG), expected MLG at equal sample size (eMLG), Shannon’s diversity index (*H*), Stoddart and Taylor’s genotypic diversity index (*G*), Simpson’s index of diversity (λ), evenness index (*E.5*), expected heterozygosity (*Hexp*), and multilocus linkage disequilibrium, measured by the index of association (*Ia*) and its standardized form (*r̄D*).

## Results

### Cluster structure with different algorithms and dimensionality reduction

**Fig. 1:**
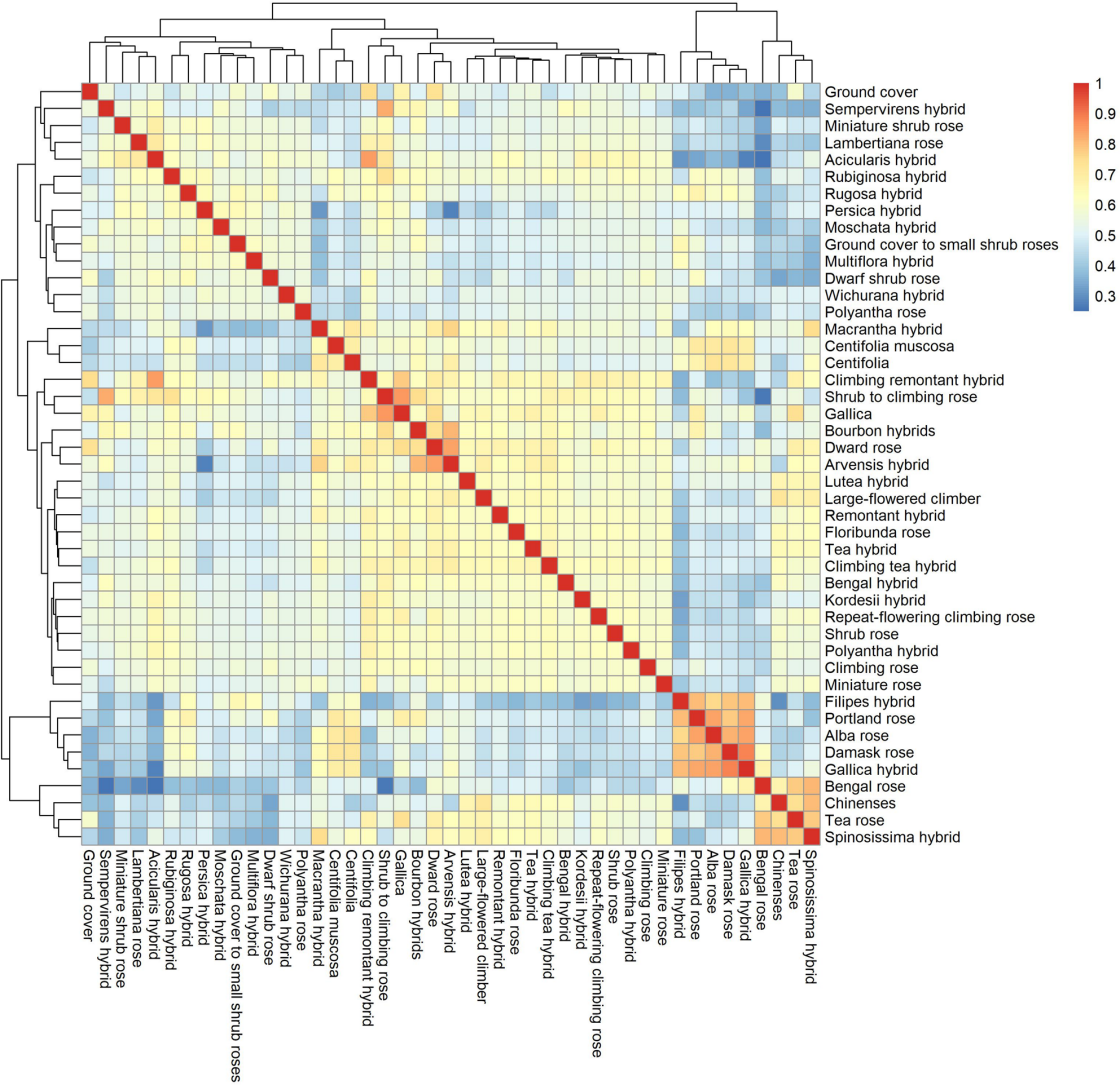
Heatmap of identity-by-state (IBS) values calculated using the SNPRelate package, with accessions aggregated by horticultural group rather than displayed individually. Rows and columns correspond to different horticultural groups, and the color scale represents the pairwise genetic similarity (IBS), ranging from low similarity (blue) to high similarity (red). The dendrograms along the axes indicate hierarchical clustering of the horticultural groups based on their genetic similarity

To explore the genetic structure and relatedness among rose horticultural groups, IBS analysis was performed using 18 SNP markers. The heatmap reveals that certain groups, such as e.g., bengal roses, *R. chinensis*, tea roses, and spinosissima hybrids exhibit high pairwise similarity, indicating closely related genetic backgrounds (Fig. 1). Similarly, filipes hybrids, portland roses, alba roses, damask roses, and gallica hybrids clustered together. In contrast, groups such as bengal roses and sempervirens hybrids are genetically more distant, forming distinct clusters in the dendrogram.

**Fig. 2:**
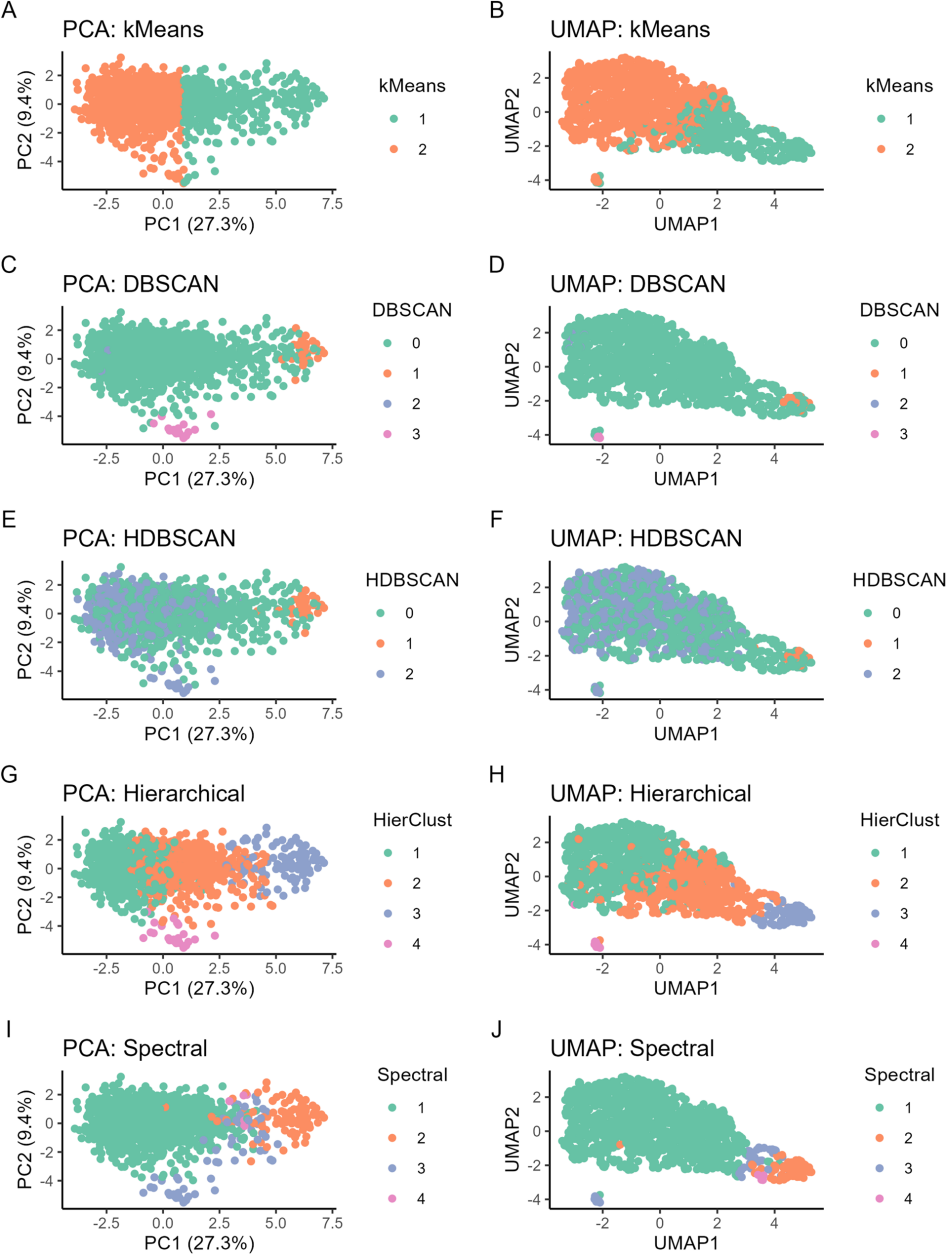
Comparison of clustering results using five algorithms on PCA- and UMAP-reduced data. Scatter plots display clustering results obtained with k-means (**A**,** B**), DBSCAN (**C**,** D**), HDBSCAN (**E**,** F**), hierarchical clustering (**G**,** H**), and spectral clustering (**I**,** J**), projected in two dimensions using principal component analysis (PCA, left column) and uniform manifold approximation and projection (UMAP, right column). The colors indicate the cluster assignments determined by each algorithm. DBSCAN and HDBSCAN include a “Cluster 0” designation for noise points. The variance explained by the first two principal components is shown on the PCA axes

To further explore the structure and variability within the SNP dataset, dimensionality reduction was performed using principal component analysis (PCA) and uniform manifold approximation and projection (UMAP). PCA revealed that the first two principal components accounted for 27.3% and 9.4% of the total variance, respectively (Fig. 2). The number and distinctiveness of the identified clusters varied substantially depending on the clustering algorithm (k-means, DBSCAN, HDBSCAN, hierarchical clustering, spectral clustering). K-means clustering yielded two clearly separated clusters, with minimal overlap between the two groups in the PCA (Fig. 2A) and UMAP space (Fig. 2B). Hierarchical clustering resolved four clusters (Fig. 2G and H), with overlap between Clusters 1 and 2, but the overall structure remained distinguishable. Spectral clustering also revealed four clusters (Fig. 2I and J), with well-separated groups in UMAP space. In contrast, density-based approaches (DBSCAN and HDBSCAN) yielded less consistent results, with the majority of the data points assigned to Cluster 0, which can be interpreted as noise. Notably, more than 350 accessions were consistently assigned to k-means Cluster 2, hierarchical Cluster 1, spectral Cluster 1, and HDBSCAN Cluster 2, indicating strong agreement across fundamentally different clustering algorithms (Additional File 3). An additional > 300 accessions were likewise assigned to k-means Cluster 2, hierarchical Cluster 1, and spectral Cluster 1, although these accessions were not classified by HDBSCAN and were instead placed into the noise category (Cluster 0).

To explore which genetic markers contributed most to the observed cluster separation, mean allele scores per cluster for each SNP were calculated for all the clustering algorithms (Additional File 4). Across all the clustering methods, several SNPs showed consistent shifts in allele frequency between clusters. For instance, RhK5_1295_1946 and RhMCRND_11585_178 strongly differentiated k-means Clusters 1 and 2, whereas RhK5_299_2775 varied markedly across hierarchical and spectral clusters. Notably, density-based methods such as DBSCAN and HDBSCAN produced more heterogeneous patterns. Interestingly, Cluster 1 in both methods was characterized by SNPs with relatively homogenous allele dosages. Despite algorithmic differences, SNPs such as RhK5_10792_6318 showed cluster-specific shifts in most methods, suggesting that these loci were broadly informative for genetic structure within the dataset. Notably, this marker is located at 66.8 Mbp on chromosome 2 [17], within the genomic interval (≈ 62–73 Mbp) repeatedly associated with fragrance and closely adjacent to loci linked to petal morphology [44, 45]. Conversely, some SNPs (e.g., RhK5_6968_582) exhibited little variation across clusters, indicating a limited contribution to group separation.

### Garden rose groups

#### Genetic clustering to investigate the shared structure between garden rose groups

**Fig. 3:**
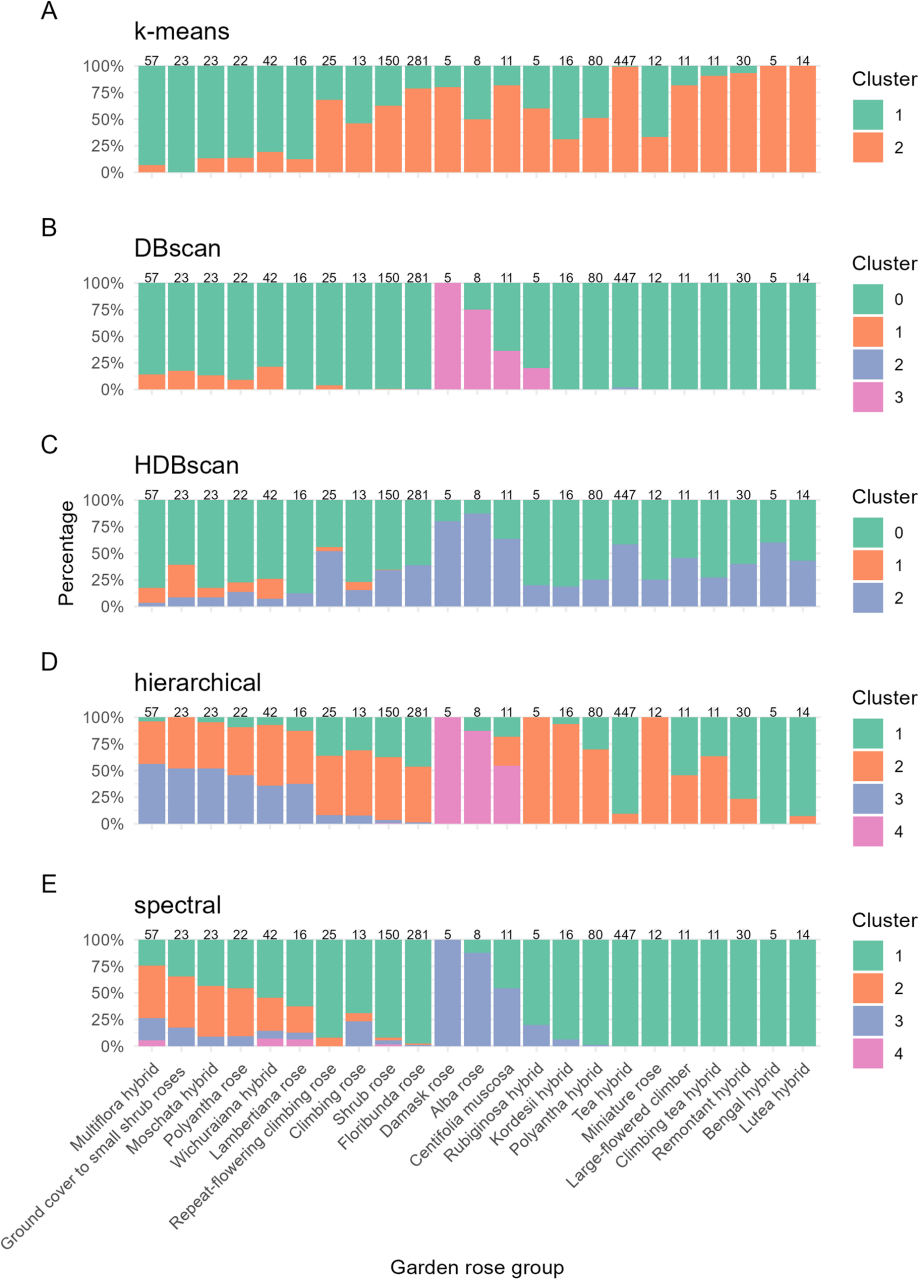
Distribution of clustering assignments across predefined rose cultivar classes. Stacked bar plots show the proportion of individuals from each cultivar class assigned to the respective clusters as identified by five clustering algorithms: K-means (**A**), DBSCAN (**B**), HDBSCAN (**C**), hierarchical clustering (**D**), and spectral clustering (**E**). Each bar represents a cultivar class (x-axis), with sample size indicated above (e.g., n = 23). The colors correspond to cluster identities

To investigate the genetic relatedness between predefined horticultural groups from the ERS, the cluster composition within each class was analyzed for rose groups with at least five accessions in our dataset (Fig. 3). Several classes displayed a high degree of homogeneity and were predominantly assigned to a single cluster across the k-means, hierarchical and spectral algorithms. For example, bengal hybrids and tea hybrids frequently co-clustered, particularly in the spectral, hierarchical, and k-means solutions, indicating close genetic relationships between these groups. The alba rose, bengal hybrids, lutea rose and tea hybrids were predominantly found in distinct clusters, with minimal admixture, suggesting unique genomic profiles. However, the bengal hybrid group comprised only five individuals, and its clustering pattern should therefore be interpreted with caution. In contrast, classes such as *centifolia muscosa* and moschata hybrids exhibited more diverse cluster affiliations, although they still tended to cooccur within shared clusters, especially when spectral and hierarchical methods were used. While density-based clustering (DBSCAN, HDBSCAN) assigned most individuals to a default cluster (Cluster 0), the few distinguishable clusters contained consistent class-specific enrichments, e.g., moschata, multiflora hybrids, polyantha rose, wichuraiana hybrids, ground cover to small shrub roses and repeat flowering climbing rose, which were grouped together in DBSCAN Cluster 1, while alba rose, *centifolia muscosa* and rubigniosa hybrids were grouped together in Cluster 3.

#### Prediction of garden rose groups using supervised learning

To assess whether the aggregation of traditional horticultural groups into genetically coherent units is meaningful, we applied supervised learning using all accessions belonging to each horticultural group, rather than restricting the analysis to accessions with an assigned cluster label. This approach allowed us to evaluate how well the newly derived cluster labels can be predicted from the genetic markers and whether they reflect consistent and biologically meaningful groupings. Based on the results of the unsupervised learning approaches, class labels were derived from the clustering outputs of k-means, hierarchical clustering, spectral clustering, DBSCAN, and HDBSCAN (Fig. 3). For each rose group in which one cluster accounted for at least 75% of the accessions, a corresponding cluster label (e.g., Cluster 1, Cluster 2, etc.) was assigned (Additional File 1), and the distributions of these grouped accessions are also visualized in PCA and UMAP space (Additional File 5). The number of accessions included in each analysis varied by method, with 542 accessions used for hierarchical clustering, 1,100 for spectral clustering, and 1,207 for HDBSCAN (see Table 2 for details on cluster size). These cluster-based labels were subsequently used to train supervised machine learning models to predict cluster membership from the 18 SNP markers. In addition, we also trained models using the original, unaggregated horticultural labels and labels derived from expert knowledge. Four algorithms were evaluated: decision trees (rpart), support vector machines with radial kernel (svmRadial), naive Bayes, and gradient boosting (xgbTree).

Original labels, as well as those derived from expert knowledge, achieved only low accuracy and/or low Cohen’s kappa values (Additional File 6). In contrast, all the labels derived from unsupervised learning achieved high accuracies, ranging from 84% to 100%, with balanced accuracies above 90% for labels derived from k-means, hierarchical, and spectral clustering (the best model selected for each label) (Table 1). The best overall performance was obtained from hierarchical and spectral clustering, which reached balanced accuracies of up to 100% and Cohen’s kappa values of up to 1.00. In the hierarchical clustering, Cluster 1 included tea hybrids, remontant hybrids, lutea hybrids, and bengal hybrids; Cluster 2 included kordesii hybrids, miniature roses, and rubiginosa hybrids; and Cluster 4 consisted of alba and damask roses (Additional File 1). Spectral clustering yielded high performance, with Cluster 1 containing bengal hybrids, climbing tea hybrids, floribunda roses, kordesii hybrids, large-flowered climbers, lutea hybrids, miniature roses, polyantha hybrids, remontant hybrids, repeat-flowering climbing roses, rubiginosa hybrids, shrub roses, and tea hybrids; and Cluster 3 containing alba and damask roses. The k-means-derived labels, including multiflora hybrids, wichuraiana hybrids, groundcover to small shrub roses, moschata hybrids, polyantha roses, and lambertiana roses in Cluster 1, and tea hybrids, floribunda roses, remontant hybrids, lutea hybrids, large-flowered climbing roses, climbing tea hybrids, *centifolia muscosa*, damask roses and bengal hybrids in Cluster 2 were best predicted with the svmRadial algorithm, with a balanced accuracy of 92% and a Cohen’s kappa value of 0.86. Consistent training accuracies indicated that the models generalize well and were unlikely to suffer from overfitting.

**Table 1:** Performance metrics of supervised learning models for Rose classes trained on SNP data using labels derived from unsupervised clustering (k-means, DBSCAN, HDBSCAN, hierarchical, and spectral clustering)

Label	Method	Accuracy	Balanced Accuracy	F1	Kappa	Accuracy Train	Kappa Train
Cluster_k-means	svmRadial	0.95	0.92	0.86	0.83	0.93	0.77
	rpart	0.82	0.80	0.61	0.50	0.88	0.59
	naive_bayes	0.94	0.87	0.83	0.80	0.94	0.78
	xgbTree	0.94	0.90	0.85	0.81	0.95	0.82
Cluster_DBSCAN	svmRadial	0.84	0.86	0.84	0.66	0.82	0.61
	rpart	0.74	0.74	0.63	0.44	0.76	0.49
	naive_bayes	0.79	0.76	0.66	0.56	0.82	0.60
	xgbTree	0.83	0.79	0.75	0.60	0.82	0.61
Cluster_HDBSCAN	svmRadial	0.95	0.80	0.63	0.60	0.95	0.63
	rpart	0.87	0.64	0.28	0.21	0.89	0.40
	naive_bayes	0.95	0.77	0.60	0.57	0.97	0.74
	xgbTree	0.95	0.71	0.52	0.49	0.96	0.69
Cluster_Hierarchical	svmRadial	1.00	1.00	1.00	1.00	0.97	0.79
	rpart	0.86	0.90	0.71	0.43	0.81	0.35
	naive_bayes	1.00	1.00	1.00	1.00	0.97	0.83
	xgbTree	0.99	0.95	0.97	0.93	0.97	0.79
Cluster_Spectral	svmRadial	1.00	1.00	1.00	1.00	1.00	0.93
	rpart	1.00	1.00	1.00	1.00	0.99	0.66
	naive_bayes	1.00	1.00	1.00	1.00	1.00	0.93
	xgbTree	1.00	1.00	1.00	0.80	1.00	0.89

For each model and label type, the overall accuracy, balanced accuracy, F1 score, and cohen’s kappa coefficient are reported alongside the corresponding training set metrics. The best-performing model for each label set was selected on the basis of the highest balanced accuracy

**Fig. 4:**
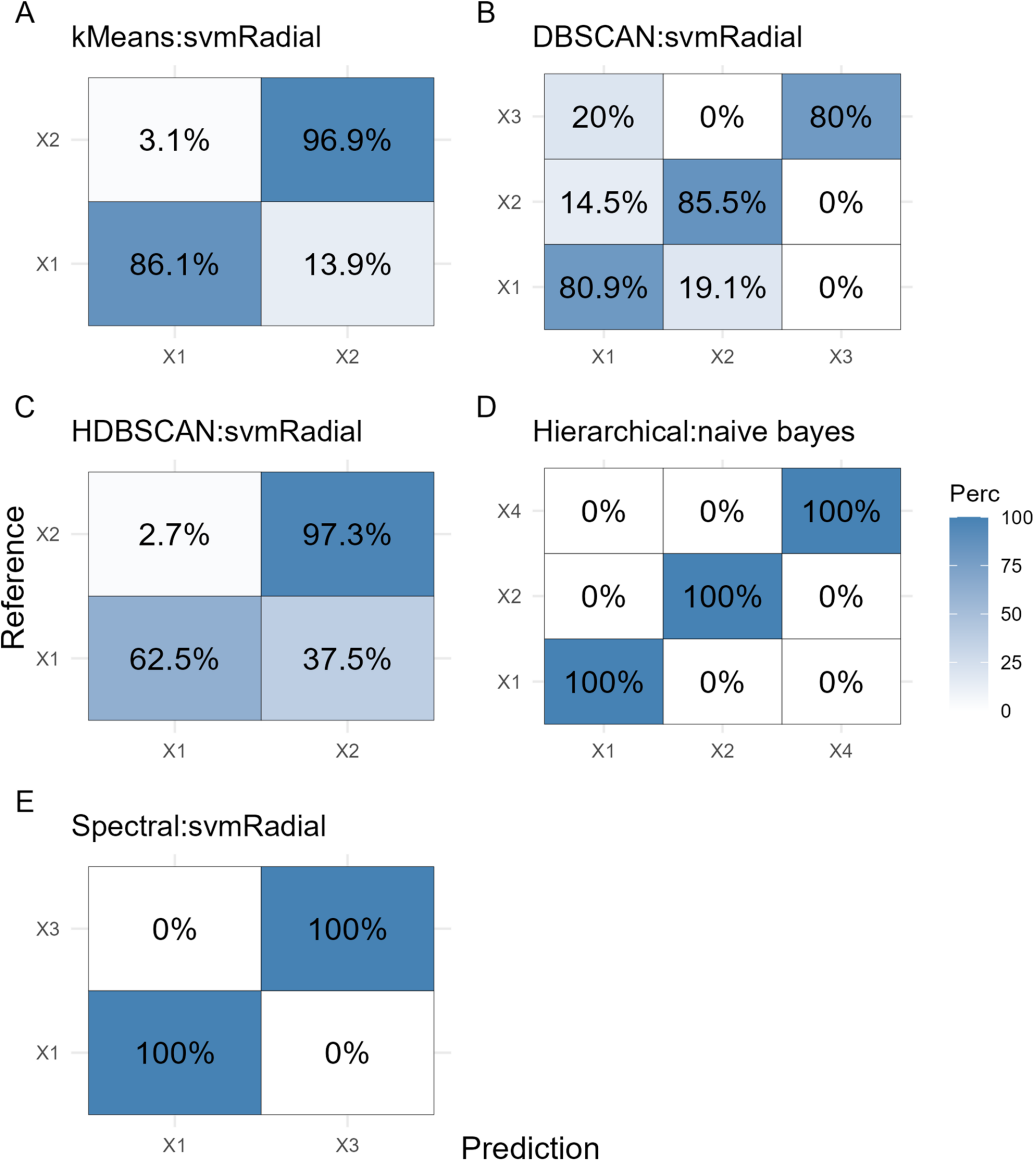
Confusion matrix displaying the classification performance of the best-performing model for a given cluster label set. The matrix shows the proportion of correctly and incorrectly predicted samples per class (in percent). The reference (true) classes are shown on the vertical axis, and the predicted classes are shown on the horizontal axis. Darker shading indicates a higher proportion of predictions within a cell. The model’s ability to distinguish between cluster labels is reflected by high values along the diagonal (correct predictions) and minimal off-diagonal misclassifications. The k-means-derived labels are Cluster 1 (X1): multiflora hybrid, wichuraiana hybrid, groundcover to small shrub roses, moschata hybrid, polyantha rose, and lambertiana rose; Cluster 2 (X2): tea hybrid, floribunda rose, remontant hybrid, lutea hybrid, large-flowered climbing rose, climbing tea hybrid, centifolia muscosa, bengal hybrid, and damask rose. The labels derived from DBSCAN are Cluster 1 (X1): ground cover to small shrub roses, moschata hybrid, multiflora hybrid, polyantha rose, repeat-flowering climbing rose, shrub rose, wichuraiana hybrid and Cluster 2 (X2): Floribunda rose, tea hybrid and Cluster 3 (X3): alba rose, centifolia muscosa, damask rose and rubiginosa hybrid. The labels derived from HDBSCAN are Cluster 1 (X1): ground cover to small shrub roses, multiflora hybrid and Cluster 2 (X2): Alba rose, bengal hybrid, centifolia muscosa, climbing tea hybrid, damask rose, floribunda rose, kordesii hybrid, lambertiana rose, large-flowered climber, lutea hybrid, miniature rose, polyantha hybrid, remontant hybrid, repeat-flowering climbing rose, rubiginosa hybrid, shrub rose, and tea hybrid. The labels derived from hierarchical clustering are Cluster 1 (X1): tea hybrid, remontant hybrid, lutea hybrid, and bengal hybrid; Cluster 2 (X2): kordesii hybrid, miniature rose, and rubiginosa hybrid; and Cluster 4 (X4): Alba and damask roses. The labels derived from spectral clustering are Cluster 1 (X1): bengal hybrid, climbing tea hybrid, floribunda rose, kordesii hybrid, large-flowered climber, lutea hybrid, miniature rose, polyantha hybrid, remontant hybrid, repeat-flowering climbing rose, rubiginosa hybrid, shrub rose, and tea hybrid; and Cluster 3 (X3): alba rose and damask rose

For each label set, the best-performing model was selected on the basis of the highest balanced accuracy, and the corresponding confusion matrix was analyzed to assess classification performance in more detail (Fig. 4). Hierarchical and spectral labels achieved perfect prediction rates of 100% across all clusters. With respect to the k-means labels, the svmRadial model correctly classified 86.1% of the samples in Cluster 1 and 96.9% in Cluster 2. In contrast, DBSCAN and HDBSCAN showed higher misclassification rates, with the HDBSCAN: svmRadial model misclassifying up to 37.5% of Cluster 1 (ground cover to small shrub roses, multiflora hybrid) samples as belonging to Cluster 2 (alba rose, bengal hybrid, *centifolia muscosa*, climbing tea, hybrid, damask rose, floribunda rose, kordesii hybrid, lambertiana rose, large-flowered climber, lutea hybrid, miniature rose, polyantha hybrid, remontant hybrid, repeat-flowering climbing rose, rubiginosa hybrid, shrub rose, and tea hybrid).

#### Genetic diversity across garden rose group clusters

To assess the biological coherence of the different clustering approaches, genetic diversity indices across clusters from different labeling methods (Additional File 1) were compared (Table 2). All the methods retained overall high genotypic diversity (λ ≈ 0.99) and expected heterozygosity values of approximately 0.65–0.70, but the distribution of diversity within clusters differed substantially.

The k-means partition separated the dataset into two clusters with clear contrasts: Cluster 1 (*n* = 183, containing multiflora hybrid, wichuraiana hybrid, groundcover to small shrub roses, moschata hybrid, polyantha rose, and lambertiana rose) showed lower diversity (H = 5.15; Hexp = 0.61) and elevated linkage disequilibrium (Ia = 2.48, r̄D = 0.146), whereas Cluster 2 (*n* = 815, containing tea hybrid, floribunda rose, remontant hybrid, lutea hybrid, large-flowered climbing rose, climbing tea hybrid, *centifolia muscosa*, bengal hybrid, and damask rose) exhibited higher diversity (H = 6.67; Hexp = 0.67) and near-random allele associations. The DBSCAN and HDBSCAN methods also yielded one large, highly diverse cluster (H = 6.56–6.98; Hexp ≈ 0.66–0.68) alongside one or more smaller groups with reduced diversity (H = 2.5–4.3; Hexp ≈ 0.33–0.61) and stronger linkage disequilibrium. The lowest diversity (H = 3.32) was reflected in Cluster 3 from DBSCAN, which contained the alba rose, centifolia muscosa, and damask rose. In the hierarchical and spectral clustering approaches, the main clusters again preserved high diversity (H = 6.16–6.95; Hexp ≈ 0.65–0.68) and very low linkage disequilibrium, whereas small clusters such as Cluster 2 in the hierarchical clustering (kordesii hybrid, miniature rose, and rubiginosa hybrid) and cluster 4, respectively 3, in both methods (alba and damask roses) displayed reduced variation (H = 2.46–3.50) and inflated disequilibrium, consistent with narrow genetic backgrounds.

**Table 2:** Genetic diversity indices for Rose accessions grouped by clusters derived from k-means, DBSCAN, HDBSCAN, hierarchical, and spectral clustering

Method	Pop	*N*	MLG	eMLG	SE	H	G	λ	E.5	Hexp	Ia	r̄D
k-means	1	183	175	175	0.000	5.15	168.3	0.994	0.977	0.610	2.48	0.146
	2	815	797	182	0.988	6.67	775.1	0.999	0.982	0.669	0.13	0.007
	Total	998	971	182	0.976	6.87	941.4	0.999	0.981	0.693	0.53	0.031
DBSCAN	1	342	329	29	0.298	5.78	317.8	0.997	0.980	0.687	1.52	0.090
	2	728	712	29	0.171	6.56	691.9	0.999	0.982	0.665	0.10	0.006
	3	29	28	28	0.000	3.32	27.1	0.963	0.981	0.539	2.82	0.166
	Total	1099	1065	29	0.160	6.96	1027.9	0.999	0.978	0.694	0.48	0.028
HDBSCAN	1	80	78	78	0.000	4.35	76.2	0.987	0.986	0.606	2.53	0.149
	2	1127	1092	80	0.445	6.98	1052.3	0.999	0.977	0.683	0.16	0.010
	Total	1207	1169	80	0.430	7.05	1126.7	0.999	0.977	0.692	0.30	0.018
Hierarchical	1	496	481	13	0.107	6.16	462.4	0.998	0.975	0.646	0.07	0.004
	2	33	33	13	0.000	3.50	33.0	0.970	1.000	0.656	0.15	0.009
	4	13	12	12	0.000	2.46	11.3	0.911	0.961	0.325	2.62	0.204
	Total	542	526	13	0.101	6.25	506.5	0.998	0.976	0.659	0.15	0.009
Spectral	1	1087	1054	13	0.071	6.95	1016.0	0.999	0.977	0.679	0.12	0.007
	3	13	12	12	0.000	2.46	11.3	0.911	0.961	0.325	2.62	0.204
	Total	1100	1066	13	0.071	6.96	1027.2	0.999	0.977	0.681	0.14	0.008

Method: clustering algorithm used (k-means or hierarchical). Pop: cluster identifier. N: number of accessions in the cluster. MLG: number of unique multilocus genotypes. eMLG: expected number of MLGs at the smallest common sample size, corrected for sample size. SE: standard error of eMLG. H: shannon’s diversity index. G: Stoddart and taylor’s genotypic diversity index. λ: simpson’s index of diversity. E.5: evenness index. Hexp: expected heterozygosity. Ia: index of association. r̄D: standardized index of multilocus linkage disequilibrium

### Breeder-specific genetic signatures

#### Genetic clustering

**Fig. 5:**
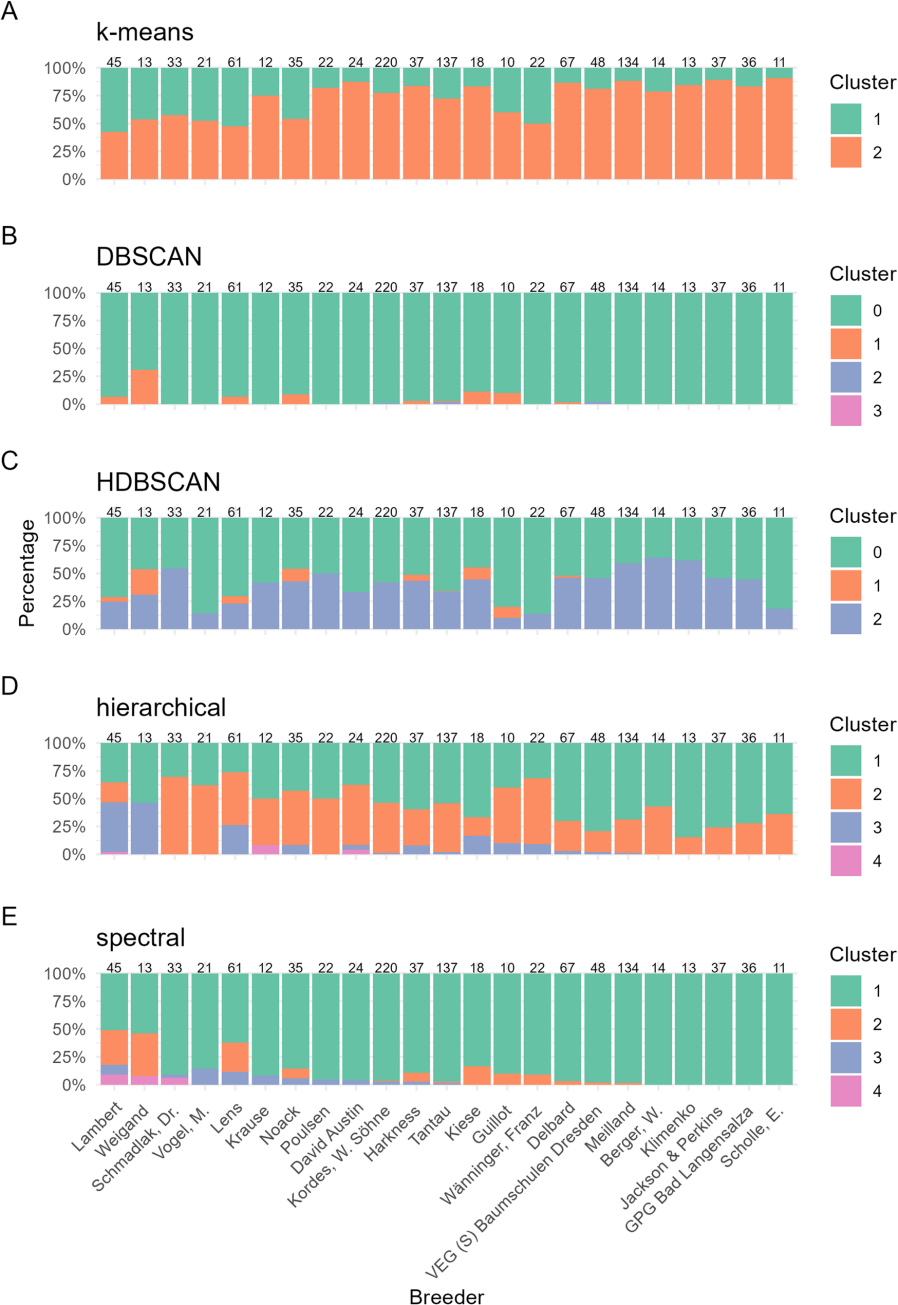
Distribution of clustering assignments across breeders of rose cultivars. Stacked bar plots show the proportion of individuals from each cultivar class assigned to the respective clusters as identified by five clustering algorithms: K-means (**A**), DBSCAN (**B**), HDBSCAN (**C**), hierarchical clustering (**D**), and spectral clustering (**E**). Each bar represents a breeder class (x-axis), with sample size indicated above (e.g., n = 23). The colors correspond to cluster identities

To evaluate whether the clustering patterns reflected the breeder-specific genetic profiles, the distribution of cluster assignments was examined for breeders with at least 10 cultivars in our dataset (Fig. 5). Several breeders showed a strong association with a single genetic cluster, particularly in the results from k-means and spectral clustering, suggesting consistent selection strategies and the use of shared parental lines within these breeding programs. For instance, the roses bred by Klimenko were predominantly assigned to Cluster 1 in the spectral and hierarchical clustering results, indicating a relatively homogeneous genetic background among their cultivars. In contrast, breeders such as Lens (cultivars in our dataset released from 1930 to 2016), Weigand (1900–1939) and Lambert (1896–1936) showed broader distributions across multiple clusters. These breeders also appeared to differ genetically from most others, as evidenced by their frequent absence from the dominant clusters occupied by the majority of other breeding programs. Of particular interest is the broad distribution of cultivars bred by Lambert, which spanned Clusters 1 to 4 in the spectral clustering results (Fig. 5E), highlighting substantial internal diversity within this breeder’s material.

Owing to genetic interconnectedness, the suitability of breeder identity as a label for supervised classification models is limited.

### Breeding years

#### Genetic clustering to assess temporal structure

**Fig. 6:**
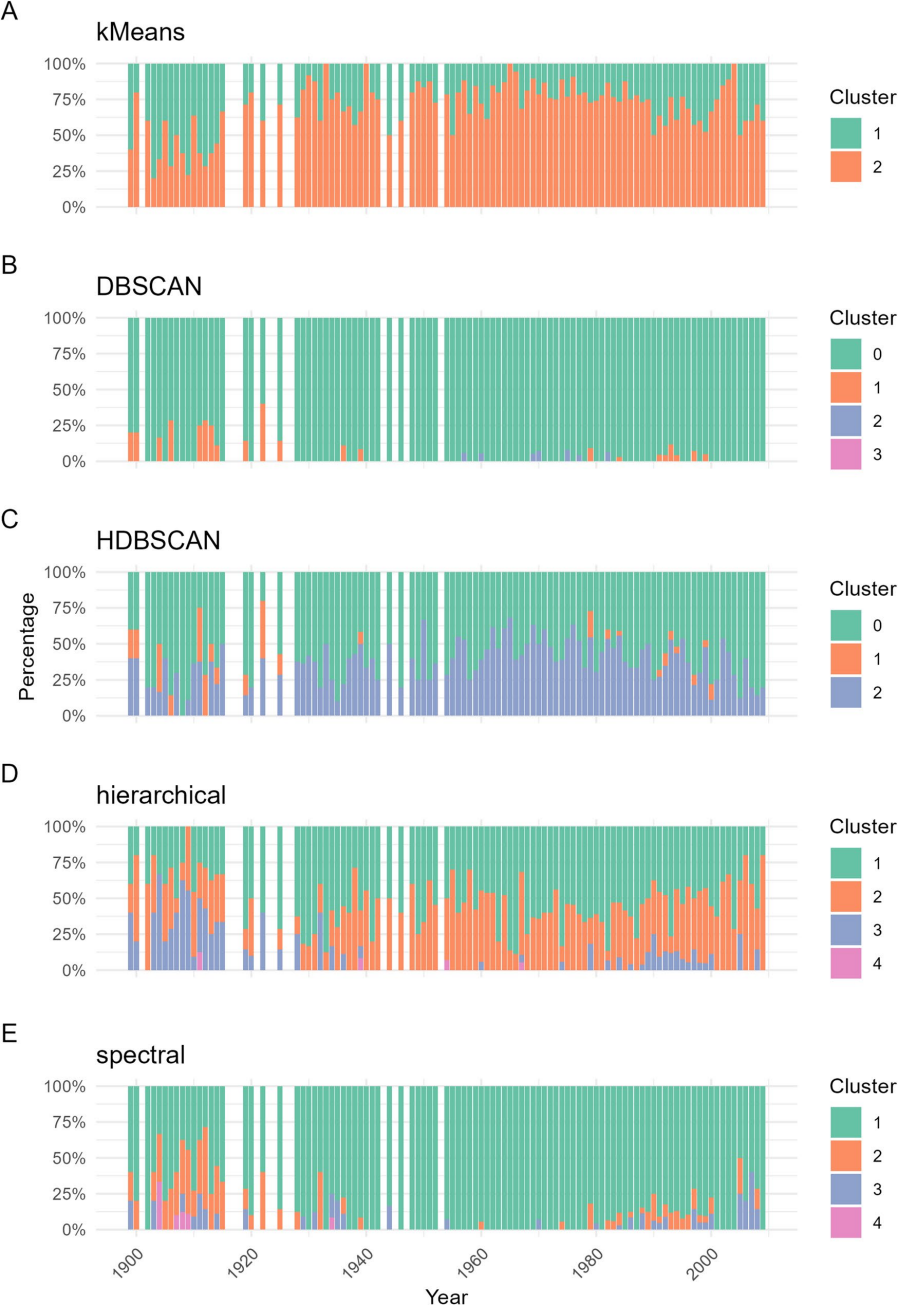
Distribution of clustering assignments across breeding years of rose cultivars. Stacked bar plots show the proportion of individuals from each cultivar class assigned to the respective clusters as identified by five clustering algorithms: K-means (**A**), DBSCAN (**B**), HDBSCAN (**C**), hierarchical clustering (**D**), and spectral clustering (**E**). Each bar represents a cultivar class (x-axis), with a sample size of at least 5. The colors correspond to cluster identities

To investigate the temporal patterns in genetic structure, the distribution of clustering assignments was analyzed across the years of cultivar release. Only data with at least 5 accessions released in the same year were analyzed. Across several clustering approaches, a noticeable shift became evident at the beginning of the 20th century (Fig. 6). In the k-means clustering, most rose cultivars released before approximately 1930 were assigned to Cluster 1, whereas after this time point, there was a pronounced increase in Cluster 2 assignments. This trend was even more evident in hierarchical clustering, where Cluster 3 was predominant among early cultivars but became less frequent in later ones. In terms of spectral clustering, Cluster 1 was primarily associated with roses released before 1930 and declined in later years. In contrast, the density-based clustering methods (DBSCAN and HDBSCAN) assigned the majority of individuals as noise (Cluster 0), limiting interpretability. Nevertheless, even in these models, a subset of early cultivars exhibited greater cluster heterogeneity, potentially indicating a more diverse and less structured gene pool in the earlier phases of rose breeding.

#### Prediction of garden rose breeding years using supervised learning

According to the results of the unsupervised learning, the samples were categorized into two groups: “roses released before 1930” (*N* = 233) and “roses released since 1930” (*N* = 1,091), which subsequently served as labels for supervised learning. Among the four supervised models tested, such as SVM, decision trees (rpart), naïve bayes and xgbTree, the support vector machine (svmRadial) and the extreme gradient boosting model (xgbTree) demonstrated the highest classification performance (Table 3). The svmRadial model achieved an accuracy of 91%, a balanced accuracy of 86%, and a Cohen’s kappa of 0.70, indicating strong agreement between the predictions and true class labels. Similarly, the xgbTree model reached an accuracy of 89%, with a balanced accuracy of 82% and a kappa value of 0.63. In particular, the training and test accuracies of the svmRadial model were comparable, implying minimal overfitting and good generalizability. The confusion matrix for the svmRadial model further highlighted its strong predictive ability for the svmRadial model, showing high true positive rates of 94% and 78.3% for both labels (Additional File 7). In contrast, the naive Bayes classifier, although yielding a high F1 score (0.92), showed a considerably lower balanced accuracy (68%) and kappa (0.43), suggesting a tendency to favor the majority class and reduced effectiveness in handling class imbalance. The decision tree model (rpart) performed the weakest overall, with a test accuracy of 86% and the lowest kappa (0.43), indicating limited predictive capability and potential overfitting.

**Table 3:** Performance metrics of supervised learning models for breeding years trained on SNP data using labels derived from unsupervised clustering (“roses released before 1930” and “roses released since 1930”).

Method	Accuracy	Balanced Accuracy	F1	Kappa	Accuracy Train	Kappa Train
svmRadial	0.91	0.86	0.95	0.70	0.85	0.51
rpart	0.84	0.79	0.90	0.51	0.81	0.42
naive_bayes	0.86	0.68	0.92	0.43	0.84	0.34
xgbTree	0.89	0.82	0.94	0.63	0.88	0.57

For each model, the overall accuracy, balanced accuracy, F1 score, and cohen’s kappa are reported alongside the corresponding training set metrics

#### Genetic diversity of roses before and after 1930

Genetic diversity indices were calculated separately for rose cultivars released before and after 1930 (Table 4). The number of unique multilocus genotypes (MLGs) was high in both groups, with 221 MLGs among 233 accessions before 1930 and 1070 MLGs among 1091 accessions after 1930. The expected number of MLGs at the smallest common sample size (eMLG) was nearly identical between groups (20.9 vs. 21.0), indicating comparable clonal richness. However, several indices suggested a notable increase in genetic diversity in cultivars released after 1930. The Shannon’s diversity index (H) increased from 5.37 to 6.97, and the Simpson’s index of diversity (λ) increased from 0.995 to 0.999, reflecting a broader genotype distribution and reduced dominance of individual clones in the modern group. Similarly, the evenness index (E.5) was slightly greater for the post-1930 cultivars (E.5 = 0.988 vs. 0.957), indicating a more evenly distributed genotype composition. The expected heterozygosity (Hexp) remained stable across time periods (0.682 before vs. 0.688 after 1930), suggesting that allelic diversity was largely maintained. In contrast, linkage disequilibrium, as measured by the index of association (Ia) and the standardized index of multilocus linkage disequilibrium (r̄D), decreased substantially in modern cultivars (Ia = 0.25 vs. 1.84; r̄D = 0.015 vs. 0.108).

**Table 4:** Summary of genetic diversity indices for Rose cultivars released before and after 1930. Pop: population group based on release date

Pop	*N*	MLG	eMLG	SE	H	G	λ	E.5	Hexp	Ia	r̄D
Pre-1930	233	221	20.9	0.339	5.37	206.4	0.995	0.957	0.682	1.84	0.108
Post-1930	1091	1070	21.0	0.088	6.97	1048.7	0.999	0.988	0.688	0.25	0.015

N: number of accessions. MLG: number of unique multilocus genotypes. eMLG: expected number of MLGs at the smallest sample size, corrected for sample size. SE: standard error of eMLG. H: shannon’s diversity index. G: Stoddart and taylor’s genotypic diversity index. λ: simpson’s index of diversity. E.5: evenness index. Hexp: expected heterozygosity. Ia: index of association (linkage disequilibrium). r̄D: standardized index of multilocus linkage disequilibrium

## Discussion

### Genetically based grouping strategies to optimize rose genebank management

The assignment of rose accessions to horticultural classes is often based on aesthetic or cultural criteria rather than genetic evidence. Unlike in crops with clear agronomic traits, ornamental plant classification is strongly shaped by historical conventions and visual appeal. In current rose genebank practices, collections are often organized independently by each institution, e.g., using different databases, leading to inconsistent label usage and a lack of standardized classification systems across repositories. Accurate assignment of roses to genetically coherent groups, while avoiding redundant or conflicting labels, is crucial for reliable estimates of genetic diversity. Such accuracy forms the basis for effective genetic resource management, the design of targeted breeding programs, and the tracing of cultivar origins.

Our results in this study confirmed that the current horticultural classification of roses does not always reflect the underlying genetic structure. While some classes, such as bengal and tea hybrids, corresponded well to distinct genetic clusters, others, e.g., ground cover to small shrub roses and wichuraiana hybrids, showed substantial heterogeneity, suggesting that visual and historical criteria alone can result in inconsistent group assignments. This finding aligns with observations from Koopmann et al. [46] and Wissemann and Ritz (2005) [3], who reported mismatches between morphological groupings and molecular-based classifications in roses. The integration of genetic and machine learning-based classification in our study provided a reproducible framework for accession grouping that could be implemented across collections.

### Interpretation of classification patterns in the context of rose taxonomy and breeding history

The genetic clustering patterns observed in this study provide important insights into how current horticultural classifications align with underlying genetic relationships. Across clustering approaches, certain cultivar groups consistently emerged as genetically distinct, whereas others appeared more closely related. Tea hybrids, remontant hybrids, lutea hybrids, and bengal hybrids were repeatedly grouped together and predicted to have high classification accuracies, indicating substantial genetic similarity among these classes. Nevertheless, within-group diversity in this cluster was relatively high (e.g., H = 6.16, hierarchical clustering), which was consistent with their complex and heterogeneous breeding histories. Similarly, alba and damask roses consistently formed a separate cluster with low internal genetic diversity (e.g., H = 2.46, hierarchical and spectral clustering), reflecting their shared historic breeding origins in Europe, and supporting the theory that they are relatives [47]. Miniature roses, kordesii hybrids, and rubiginosa hybrids also tended to cluster together, suggesting overlapping genetic backgrounds despite differing horticultural categories; their low within-group diversity (e.g., H = 3.50, hierarchical clustering) further pointed to limited genetic bases. These patterns were most pronounced in hierarchical and spectral clustering (balanced accuracy 100%) and largely reproduced in k-means (balanced accuracy 92%), indicating that the observed groupings were robust across both unsupervised and supervised classification frameworks.

Despite some breeder-specific clustering, substantial overlap in cluster membership was observed across the breeding programs in our analysis. This widespread sharing of genetic clusters indicated the repeated use of elite lines and common progenitors across breeding histories, a pattern consistent with the interconnected nature of rose breeding over the past centuries. In contrast, Vukosavljev et al. [48] reported differences between breeders, but the results strongly correlated with garden rose types. Importantly, our results are also consistent with the idea that some breeders historically specialized in particular horticultural types, while others contributed a more diverse set of cultivars, at least from the perspective of the genebank material available today. Such differences in breeding focus could naturally shape the representation of genotypic clusters in our dataset, and may therefore contribute to the observed patterns alongside the shared use of elite parental lines.

Temporal analysis of cultivar release decades revealed a pronounced shift in the genetic structure of cultivated roses around 1930, which was evident in both cluster analyses and supervised learning results, with accuracies of up to 91%. This period coincided with several pivotal developments in rose breeding history: Floribunda roses were introduced from polyantha × tea hybrids, and miniature roses originated from the rediscovery of *R. roulettii* in 1920 [49]. Accessions released before 1930 formed a more diverse range of clusters, reflecting a broad genetic base and varied breeding origins. In contrast, post-1930 cultivars increasingly coalesced into slightly fewer, genetically similar clusters, suggesting a slight bottleneck effect driven by selection and dissemination of elite parental lines throughout the 20th century. In major cereal crops such as bread wheat, allelic diversity decreased by up to 60% during domestication and improvement phases [50]. In comparison to cereals, rose breeding historically appeared to be less stringent in selection pressure, often favoring aesthetic and horticultural traits over agronomic uniformity. This phenomenon was reflected in the relative stability of expected heterozygosity (Hexp) in roses. However, notable shifts in linkage disequilibrium patterns (Ia from 1.84 to 0.25; r̄D from 0.108 to 0.015) indicated structural reorganization of the gene pool, potentially reducing the effective population size and increasing vulnerability to genetic drift and inbreeding, a phenomenon that has been well documented in small or heavily selected populations. Thus, older germplasm emerges as a vital reservoir of genetic diversity, offering valuable alleles for future breeding. This pattern might also reflect the composition of the sampled material: genebank collections might prioritize older cultivars that are maximally distinct from each other, whereas many modern cultivars tend to be more closely related, potentially amplifying the observed contrast in genetic structure between temporal groups.

### Utility and limitations of a reduced SNP marker set for rose classification and diversity assessment

The use of only 18 PCR-based SNP markers in this study represents a deliberate trade-off between resolution and practicality. While genotyping-by-sequencing (GBS) and SNP chip–based approaches in roses [51, 52] enable access to tens of thousands of loci, and thus allow a more comprehensive capture of genomic variation, their cost, technical requirements, and data processing demands can limit their routine application in genebank management. The 18 SNP marker set used in this study has been successfully applied in roses for cultivar identification [17] and offers a more affordable, rapid, and scalable solution. DNA isolation is straightforward because of the robustness of the KASP/PACE assays even when the DNA is not perfectly pure, allowing PCR genotyping to be implemented in standard molecular laboratories with minimal effort and without specialized equipment. The SNP markers used were carefully selected to be uncorrelated, eliminating the need for attribute trimming and ensuring a robust genetic basis for classification. Nevertheless, the reduced marker density constrained the differentiation power, particularly among closely related cultivars or within breeding lines sharing a narrow genetic base. This limitation is especially relevant in ornamental species such as roses, where historical breeding practices and the recurrent use of elite parents have led to highly interrelated germplasms [53]. It should be noted that the diversity and linkage disequilibrium indices presented in this study (H, G, λ, Hexp, Ia, r̄D) are based on only 18 SNP markers, which provides only a coarse approximation of genome-wide variation. As a consequence, estimates of LD reflect broad trends rather than fine-scale patterns, and the differentiation among closely related accessions may be underestimated. Despite these limitations, the indices are still informative for assessing population-level structure and for evaluating the homogeneity of the clusters identified.

In our dataset, the imbalance of group sizes added an additional challenge: several horticultural classes were represented by disproportionately few accessions, whereas others contained several hundred. Such imbalance can bias clustering and classification outcomes, as algorithms tend to be driven by patterns in the largest groups. To address this issue, we verified unsupervised clustering results using supervised learning approaches, applying synthetic minority oversampling (SMOTE) and evaluating models with balanced accuracy to mitigate the influence of dominant classes [54, 55]. Nevertheless, the unusually high to perfect classification performance obtained for hierarchical and spectral clustering can largely be explained by the extreme imbalance of the derived cluster labels. In these methods, a single dominant cluster contained the majority of accessions, while some minority clusters comprised only a few individuals. Such configurations can artificially inflate performance metrics, even when biological separability is limited, because the classifier mainly needs to distinguish one large, genetically coherent group from several very small ones. Applying the same marker panel to a more balanced dataset may yield further insights into the robustness of the observed patterns, and could help to inform future revisions of rose classifications.

## Conclusion

Even with a small set of 18 SNP markers, we achieved differentiation of major rose classes and breeding eras. These findings demonstrated that cost-effective, low-density SNP panels could be applied not only for cultivar fingerprinting but also for diversity evaluation in large plant genetic resource collections. Importantly, such standardized genetic data could serve as a foundation for label harmonization across institutions, facilitating interoperability between genebanks and supporting long-term conservation strategies. For practical harmonization of additional collections with this 18 marker panel, we recommend a hierarchical labelling strategy that preserves horticultural classes at the coarse level and incorporates the genetically coherent aggregate groups identified in this study (e.g., the asian hybrid aggregate comprising tea, remontant, lutea and bengal hybrids; the alba–damask lineage; and the miniature–kordesii–rubiginosa cluster) as an intermediate level, while assigning consensus-derived cluster IDs at the fine level. Practically, we suggest using PCA and UMAP for quality control, applying k-means as the primary clustering method, supported by Ward hierarchical and spectral clustering, and adopting a consensus rule (e.g., ≥ 70% agreement across methods) to define robust fine-level labels. For routine scaling to new collections, supervised classifiers such as svmRadial may be trained on the consensus-labelled core set.

## Supplementary Information

Below is the link to the electronic supplementary material.

Supplementary Material 1. SNP dataset with cultivar labels and unsupervised clustering assignments. The SNP marker panel was adopted from Patzer et al. [17]. The cultivar labels were provided by the Europa-Rosarium Sangerhausen. Unsupervised clustering assignments were added, with samples assigned to a cluster when at least 75% of group membership was consistent with a predominant cluster

Supplementary Material 2. Hierarchical clustering dendrogram based on SNP dosage data. The dendrogram was generated using hierarchical clustering with Ward’s method (ward.D2). Each leaf represents one accession, and branch lengths reflect pairwise genetic dissimilarity among samples. The cut height corresponding to k = 4 clusters is indicated by a horizontal line.

Supplementary Material 3. Upset plot illustrating the intersections of horticultural groups identified across the different clustering methods. Each vertical bar represents the size of an intersection between groups, while the connected dots below the bars specify which combinations of methods contribute to that particular intersection.

Supplementary Material 4. Cluster-specific SNP mean values across the five clustering algorithms. Heatmaps display the mean allele values for each SNP (rows) across clusters (columns) as determined by k-means, DBSCAN, HDBSCAN, hierarchical clustering, and spectral clustering. The color intensity reflects the mean allele score per SNP per cluster, from 0 (yellow) to 4 (red). DBSCAN and HDBSCAN include cluster “0”, which represents unassigned (noise) points.

Supplementary Material 5. Visualization of the horticultural groups projected into PCA and UMAP space based on the dominant unsupervised-learning clusters. For each horticultural class, the cluster most frequently assigned to its members (dominant cluster) was identified, and subsequently assigned all accessions of that class to this cluster.

Supplementary Material 6. Performance metrics of supervised learning models for rose classes trained on SNP data using original horticultural labels and labels derived on expert knowledge.

Supplementary Material 7. Confusion matrix displaying the classification performance of the svmRadial Model for roses bred before and after 1930. The matrix shows the proportion of correctly and incorrectly predicted samples per class (in percents). The reference (true) classes are shown on the vertical axis, and the predicted classes are shown on the horizontal axis. Darker shading indicates a higher proportion of predictions within a cell. The model’s ability to distinguish between cluster labels is reflected by high values along the diagonal (correct predictions) and minimal off-diagonal misclassifications.

## Data Availability

All data generated or analysed during this study are included in this published article and its supplementary information files. The R code used in this study is publicly available on Zenodo (DOI: 10.5281/zenodo.18140823).

## Abbreviations

E.5: Evenness Index
eMLG: Expected MLG at Equal Sample Size
ERS: Europa Rosarium Sangerhausen
G: Stoddart and Taylor’s Genotypic Diversity Index
H: Shannon’s Diversity Index
Hexp: Expected Heterozygosity
Ia: Index of Association
ML: Machine Learning
MLG: Multilocus Genotype
PACE: PCR Allele Competitive Extension
PCA: Principal Component Analysis
SMOTE: Synthetic Minority Over-sampling Technique
SNP: Single Nucleotide Polymorphism
SVM: Support Vector Machine
UMAP: Uniform Manifold Approximation and Projection
λ: Simpson’s Diversity Index
