# Supplementary figures and images for: Machine learning-based classification of roses using 18 SNP markers for optimized genebank management
Source: Plant Methods. 2026 Jan 6;22:8. doi: 10.1186/s13007-025-01496-0 (PMC12849561; doi:10.1186/s13007-025-01496-0)

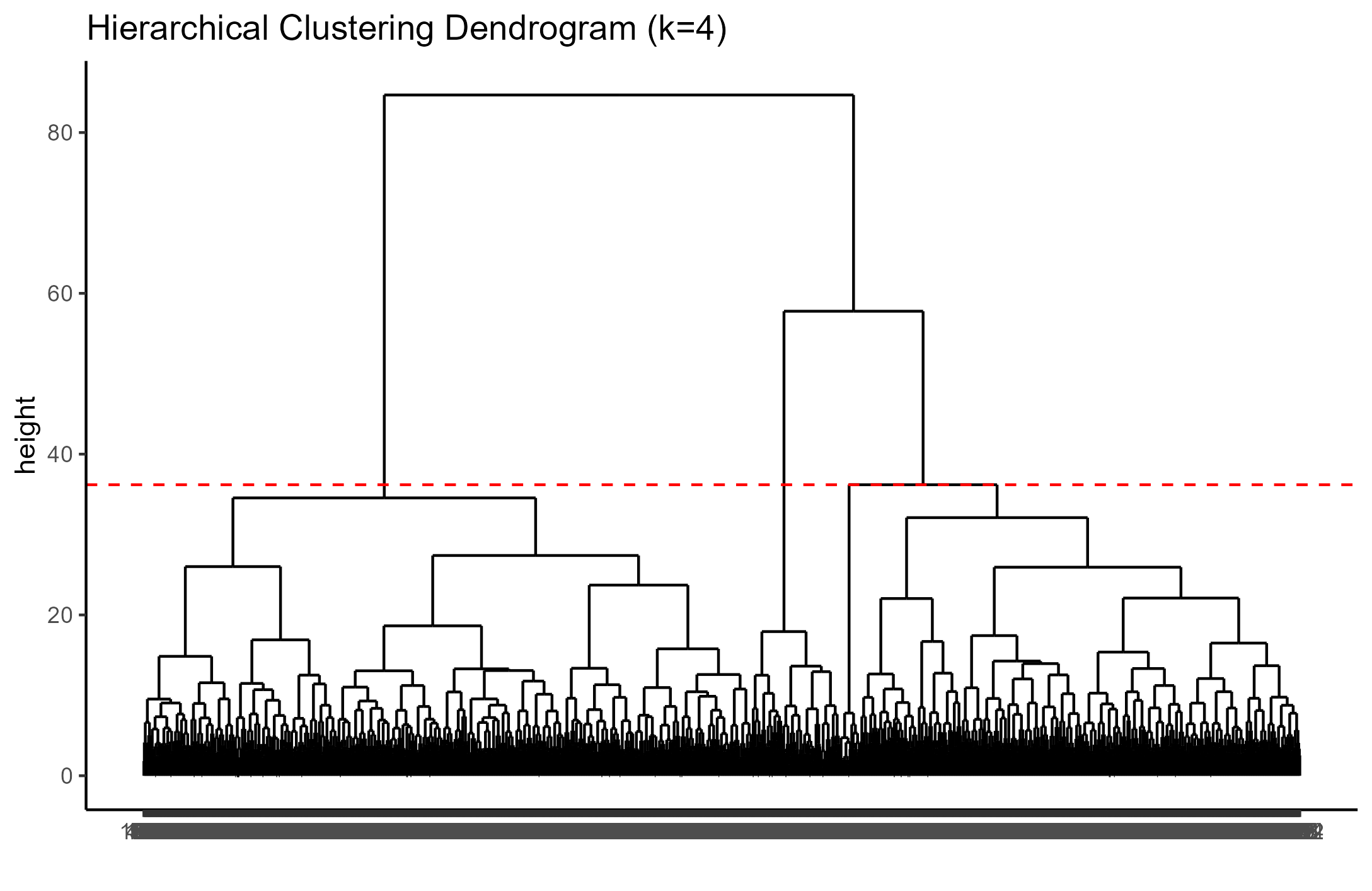

Supplement: Supplementary file 2 — Supplementary Material 2. Hierarchical clustering dendrogram based on SNP dosage data. The dendrogram was generated using hierarchical clustering with Ward’s method (ward.D2). Each leaf represents one accession, and branch lengths reflect pairwise genetic dissimilarity among samples. The cut height corresponding to k = 4 clusters is indicated by a horizontal line. [file 13007_2025_1496_MOESM2_ESM.png]

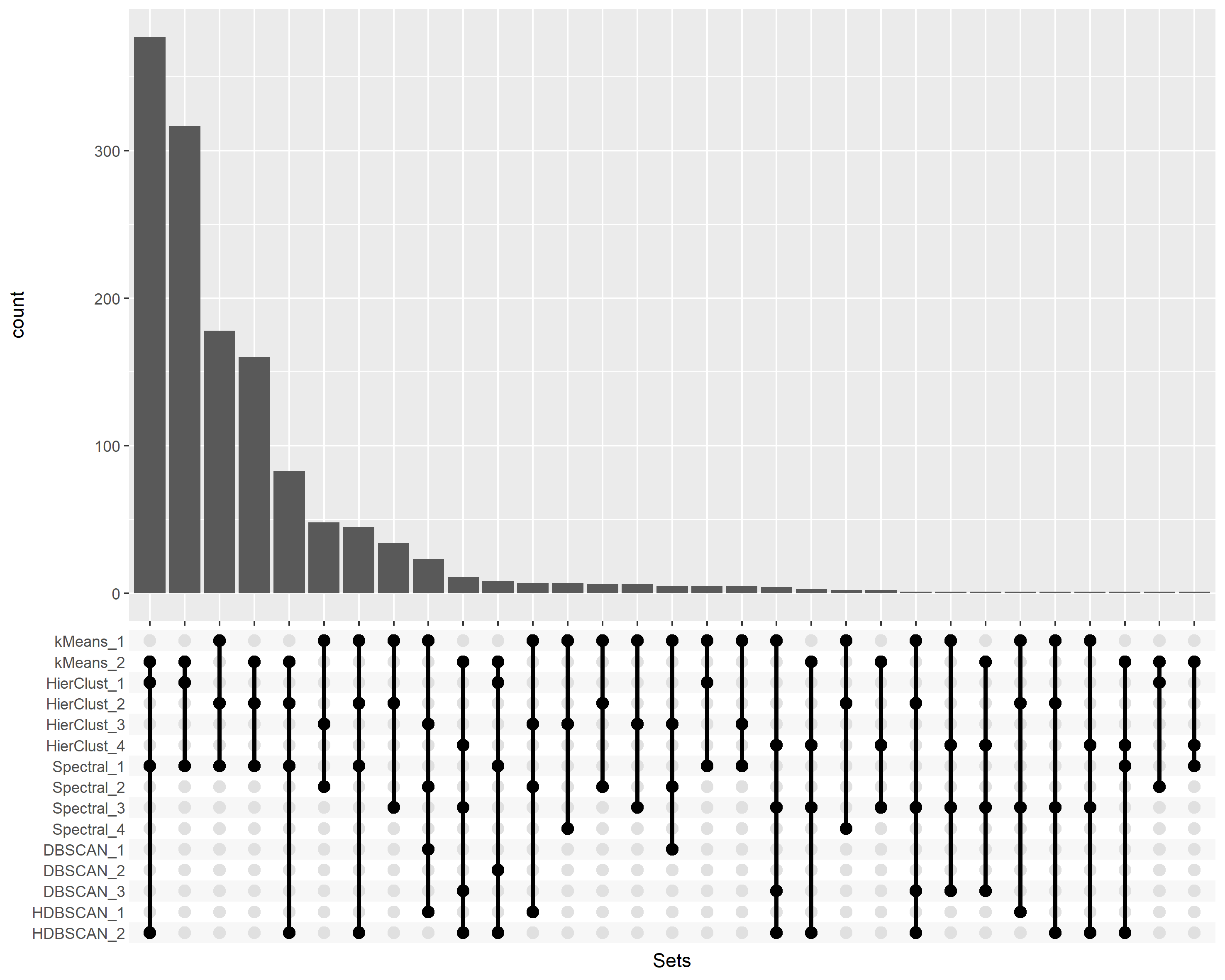

Supplement: Supplementary file 3 — Supplementary Material 3. Upset plot illustrating the intersections of horticultural groups identified across the different clustering methods. Each vertical bar represents the size of an intersection between groups, while the connected dots below the bars specify which combinations of methods contribute to that particular intersection. [file 13007_2025_1496_MOESM3_ESM.png]

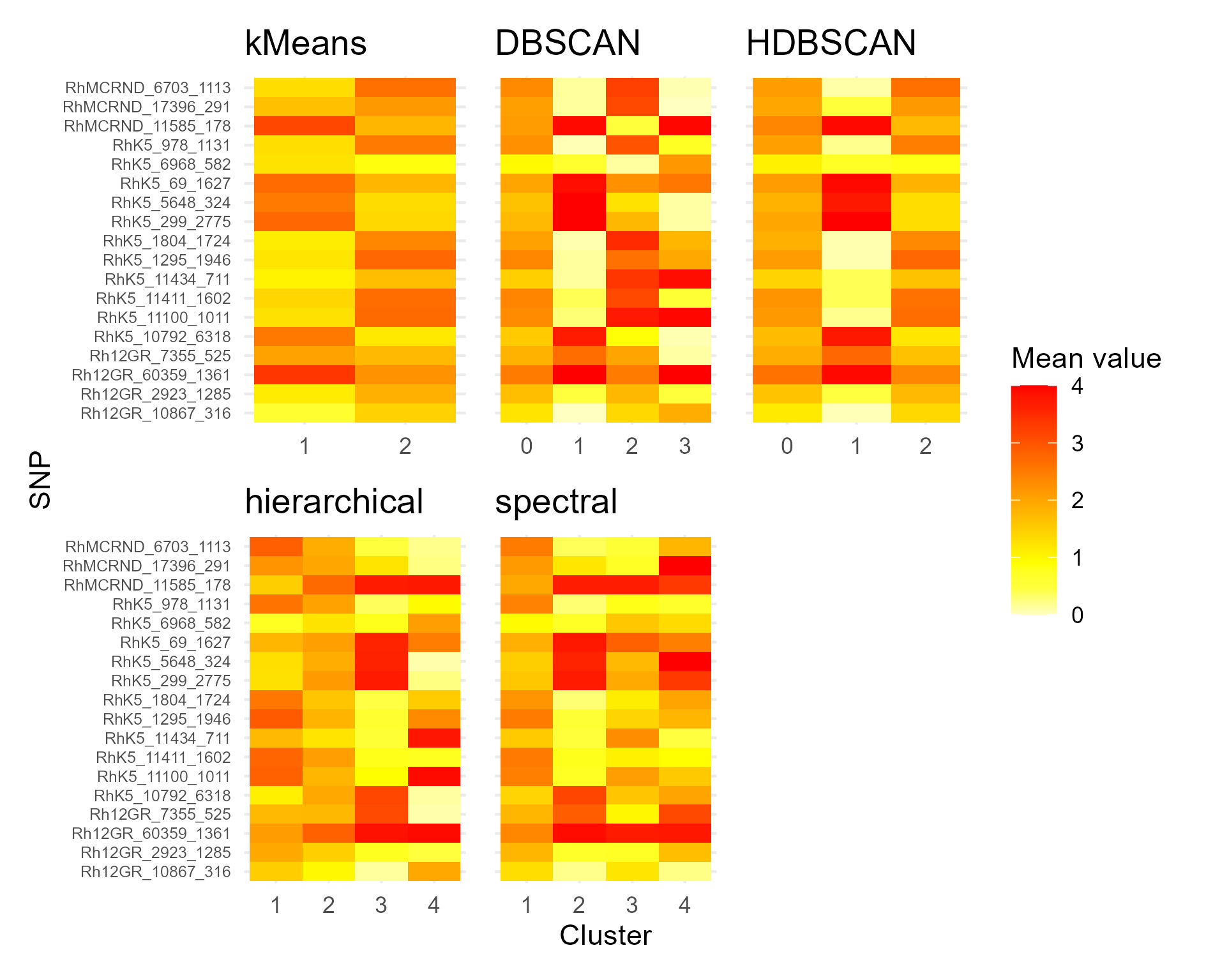

Supplement: Supplementary file 4 — Supplementary Material 4. Cluster-specific SNP mean values across the five clustering algorithms. Heatmaps display the mean allele values for each SNP (rows) across clusters (columns) as determined by k-means, DBSCAN, HDBSCAN, hierarchical clustering, and spectral clustering. The color intensity reflects the mean allele score per SNP per cluster, from 0 (yellow) to 4 (red). DBSCAN and HDBSCAN include cluster “0”, which represents unassigned (noise) points. [file 13007_2025_1496_MOESM4_ESM.jpg]

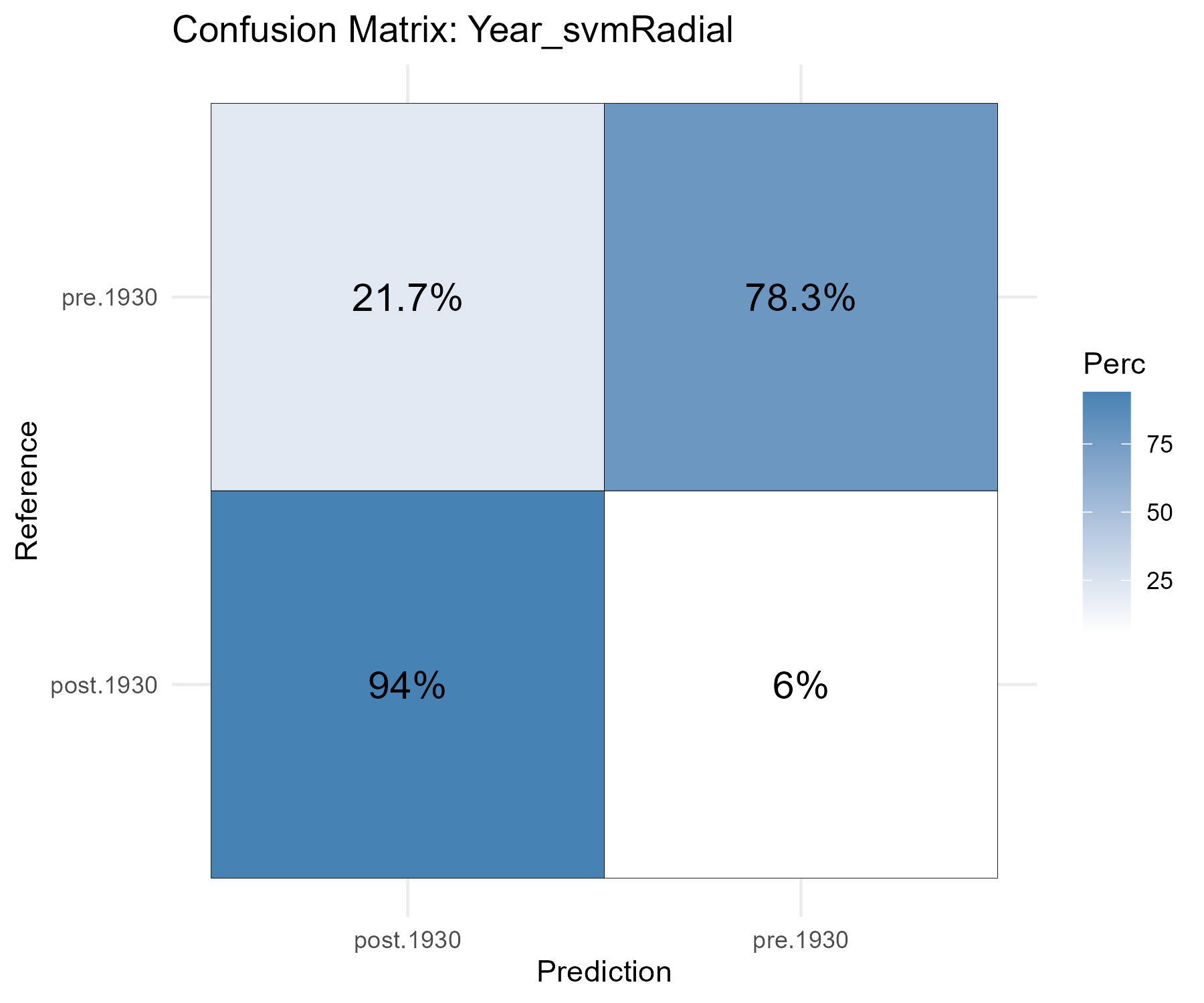

Supplement: Supplementary file 7 — Supplementary Material 7. Confusion matrix displaying the classification performance of the svmRadial Model for roses bred before and after 1930. The matrix shows the proportion of correctly and incorrectly predicted samples per class (in percents). The reference (true) classes are shown on the vertical axis, and the predicted classes are shown on the horizontal axis. Darker shading indicates a higher proportion of predictions within a cell. The model’s ability to distinguish between cluster labels is reflected by high values along the diagonal (correct predictions) and minimal off-diagonal misclassifications. [file 13007_2025_1496_MOESM7_ESM.png]
