# Supplementary material for: Machine learning-based classification of roses using 18 SNP markers for optimized genebank management
Source: Plant Methods. 2026 Jan 6;22:8. doi: 10.1186/s13007-025-01496-0 (PMC12849561; doi:10.1186/s13007-025-01496-0)
Supplement: Supplementary file 5 — Supplementary Material 5. Visualization of the horticultural groups projected into PCA and UMAP space based on the dominant unsupervised-learning clusters. For each horticultural class, the cluster most frequently assigned to its members (dominant cluster) was identified, and subsequently assigned all accessions of that class to this cluster. [file 13007_2025_1496_MOESM5_ESM.pdf]

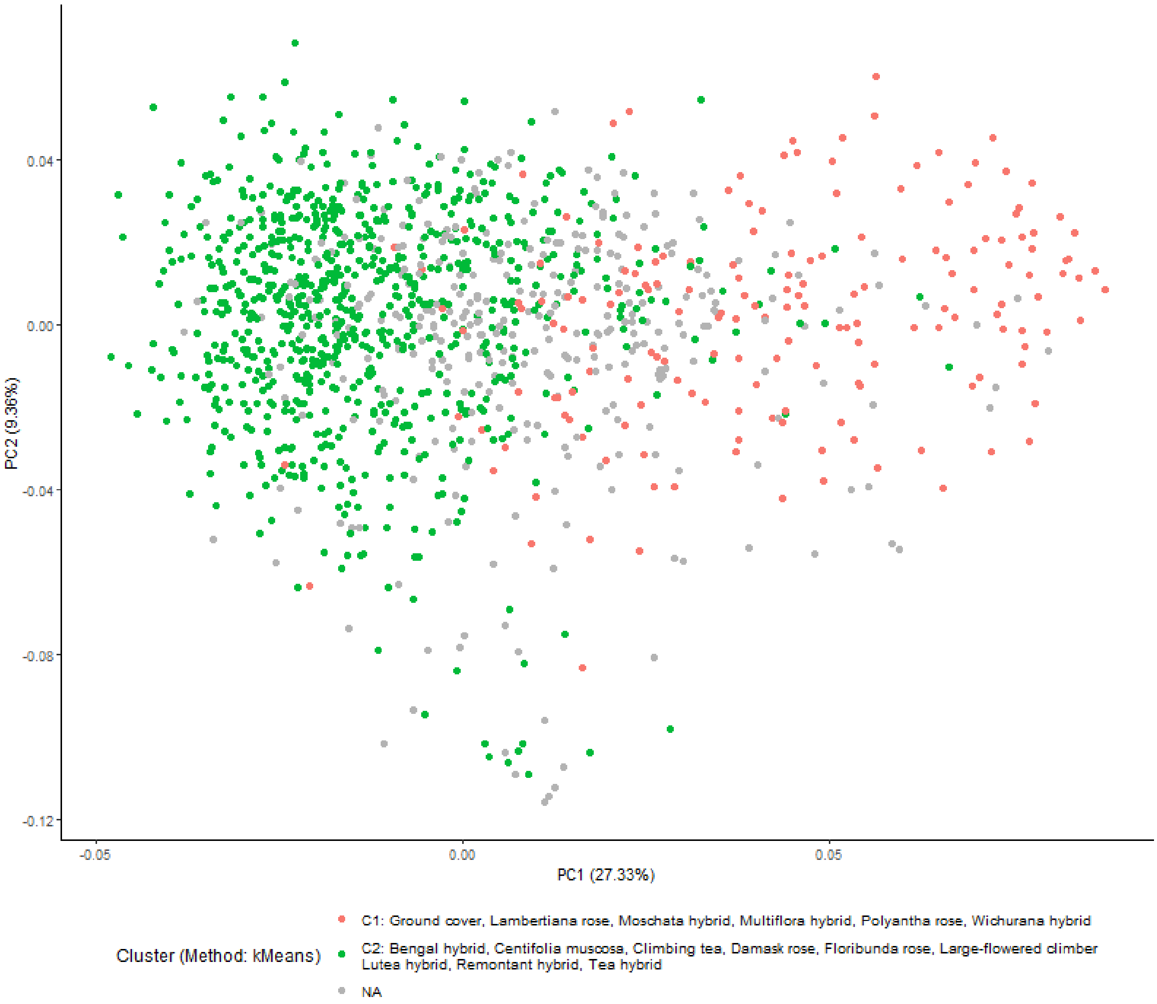

0.04
0.00
-0.04
-0.08
-0.12
PC2 (9.36%)
-0.05
0.00
0.05
PC1 (27.33%)
Cluster (Method: kMeans)
C1: Ground cover, Lambertiana rose, Moschata hybrid, Multiflora hybrid, Polyantha rose, Wichurana hybrid
C2: Bengal hybrid, Centifolia muscosa, Climbing tea, Damask rose, Floribunda rose, Large-flowered climber Lutea hybrid, Remontant hybrid, Tea hybrid
NA

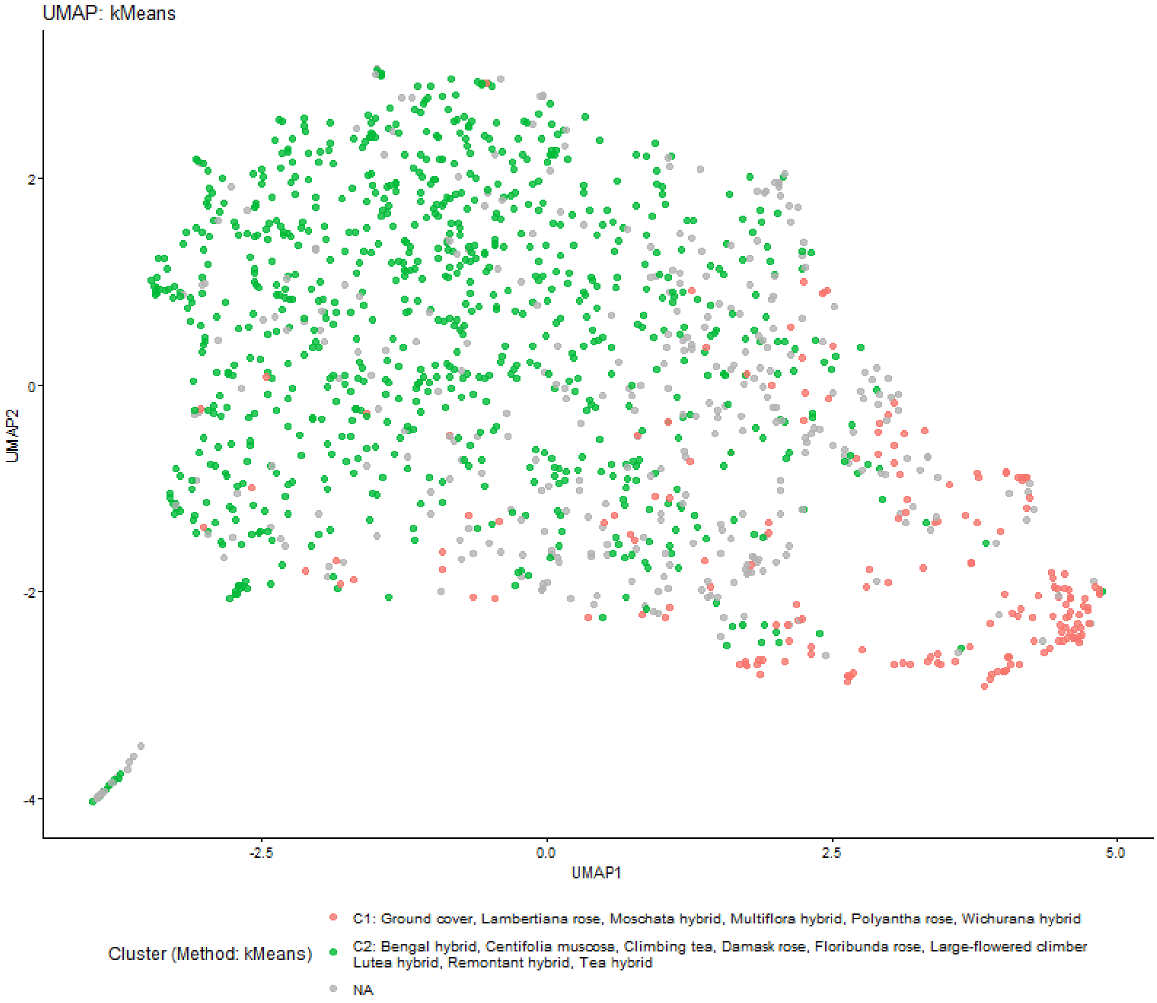

UMAP: kMeans
2
0
-2
-4
UMAP2
-2.5
0.0
2.5
5.0
UMAP1
Cluster (Method: kMeans)
C1: Ground cover, Lambertiana rose, Moschata hybrid, Multiflora hybrid, Polyantha rose, Wichurana hybrid
C2: Bengal hybrid, Centifolia muscosa, Climbing tea, Damask rose, Floribunda rose, Large-flowered climber Lutea hybrid, Remontant hybrid, Tea hybrid
NA

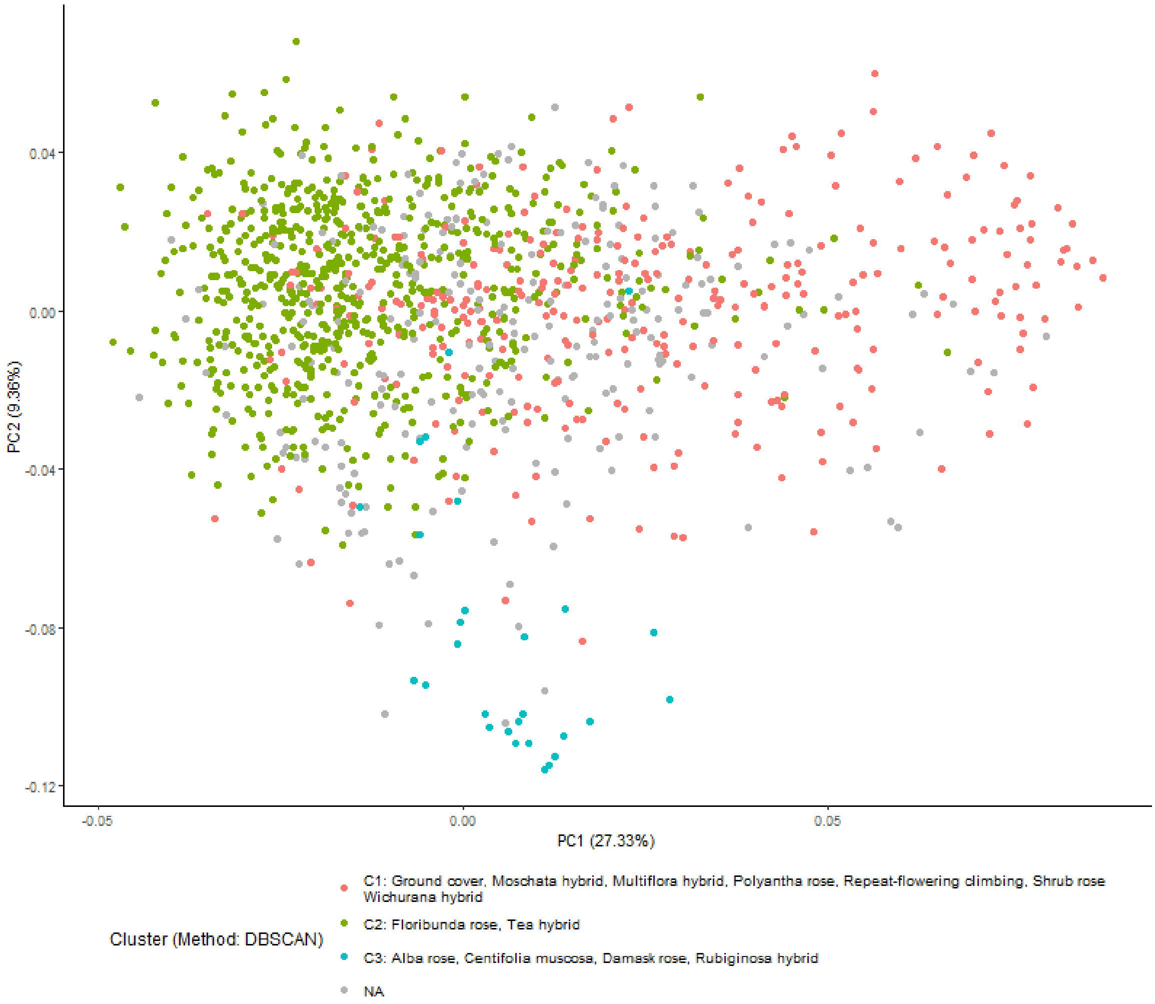
PC2 (9.36%)
0.04
0.00
-0.04
-0.08
-0.12
-0.05
0.00
0.05
PC1 (27.33%)
Cluster (Method: DBSCAN)
C1: Ground cover, Moschata hybrid, Multiflora hybrid, Polyantha rose, Repeat-flowering climbing, Shrub rose Wichurana hybrid
C2: Floribunda rose, Tea hybrid
C3: Alba rose, Centifolia muscosa, Damask rose, Rubiginosa hybrid
NA

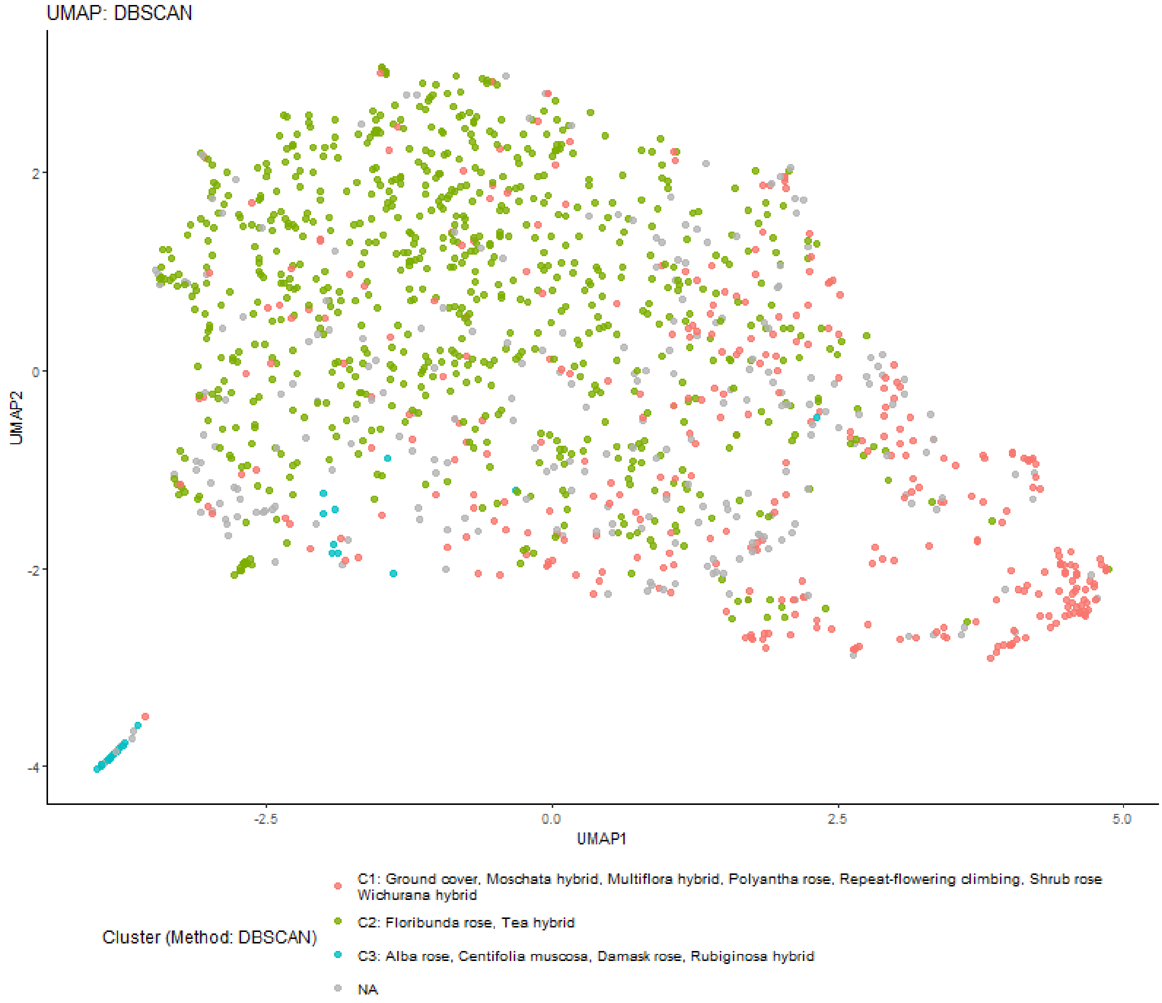
UMAP: DBSCAN
UMAP2
2
0
-2
-4
-2.5
0.0
2.5
5.0
UMAP1
Cluster (Method: DBSCAN)
C1: Ground cover, Moschata hybrid, Multiflora hybrid, Polyantha rose, Repeat-flowering climbing, Shrub rose Wichurana hybrid
C2: Floribunda rose, Tea hybrid
C3: Alba rose, Centifolia muscosa, Damask rose, Rubiginosa hybrid
NA

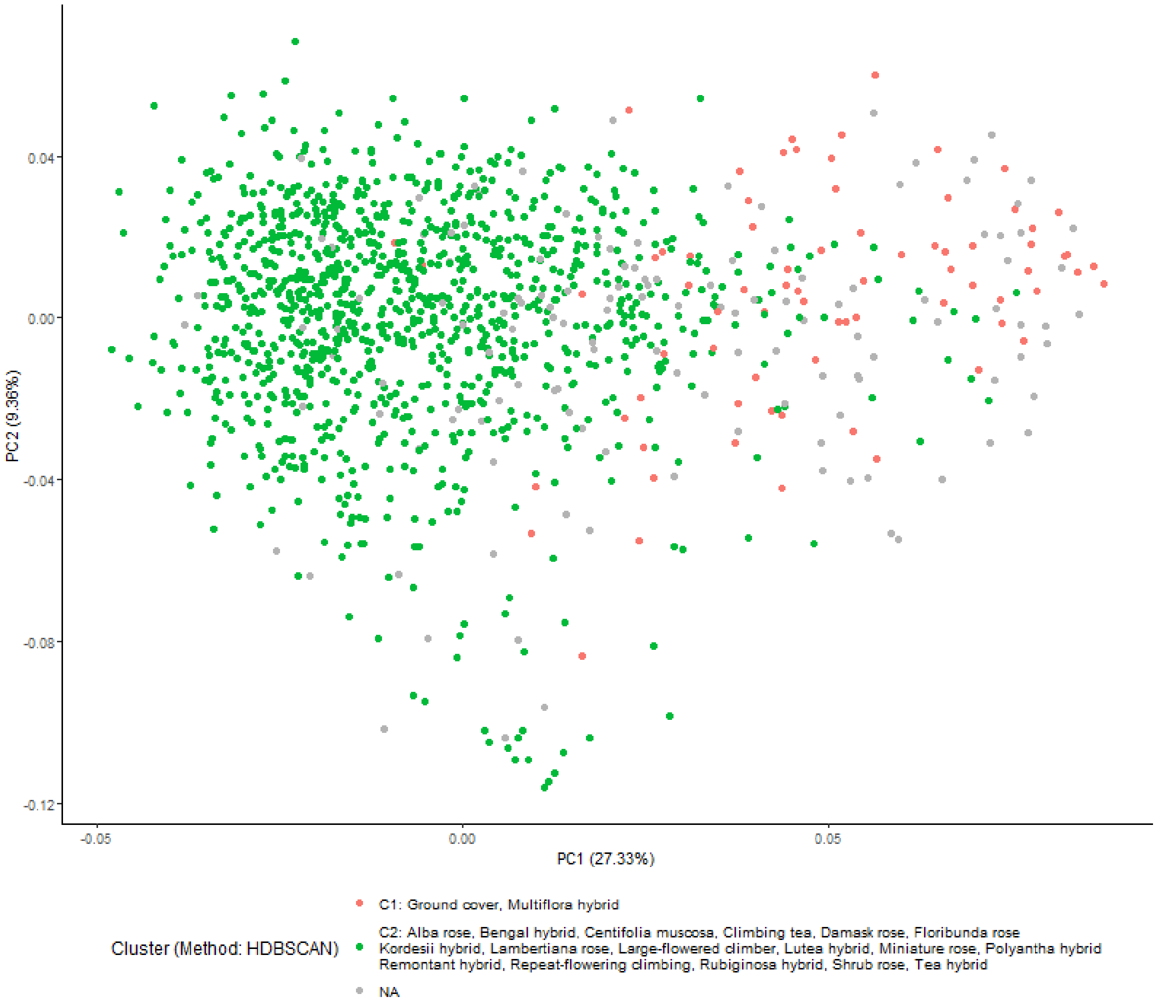

PC2 (9.36%)
0.04
0.00
-0.04
-0.08
-0.12
-0.05
0.00
0.05
PC1 (27.33%)
Cluster (Method: HDBSCAN)
C1: Ground cover, Multiflora hybrid
C2: Alba rose, Bengal hybrid, Centifolia muscosa, Climbing tea, Damask rose, Floribunda rose Kordesii hybrid, Lambertiana rose, Large-flowered climber, Lutea hybrid, Miniature rose, Polyantha hybrid Remontant hybrid, Repeat-flowering climbing, Rubiginosa hybrid, Shrub rose, Tea hybrid
NA

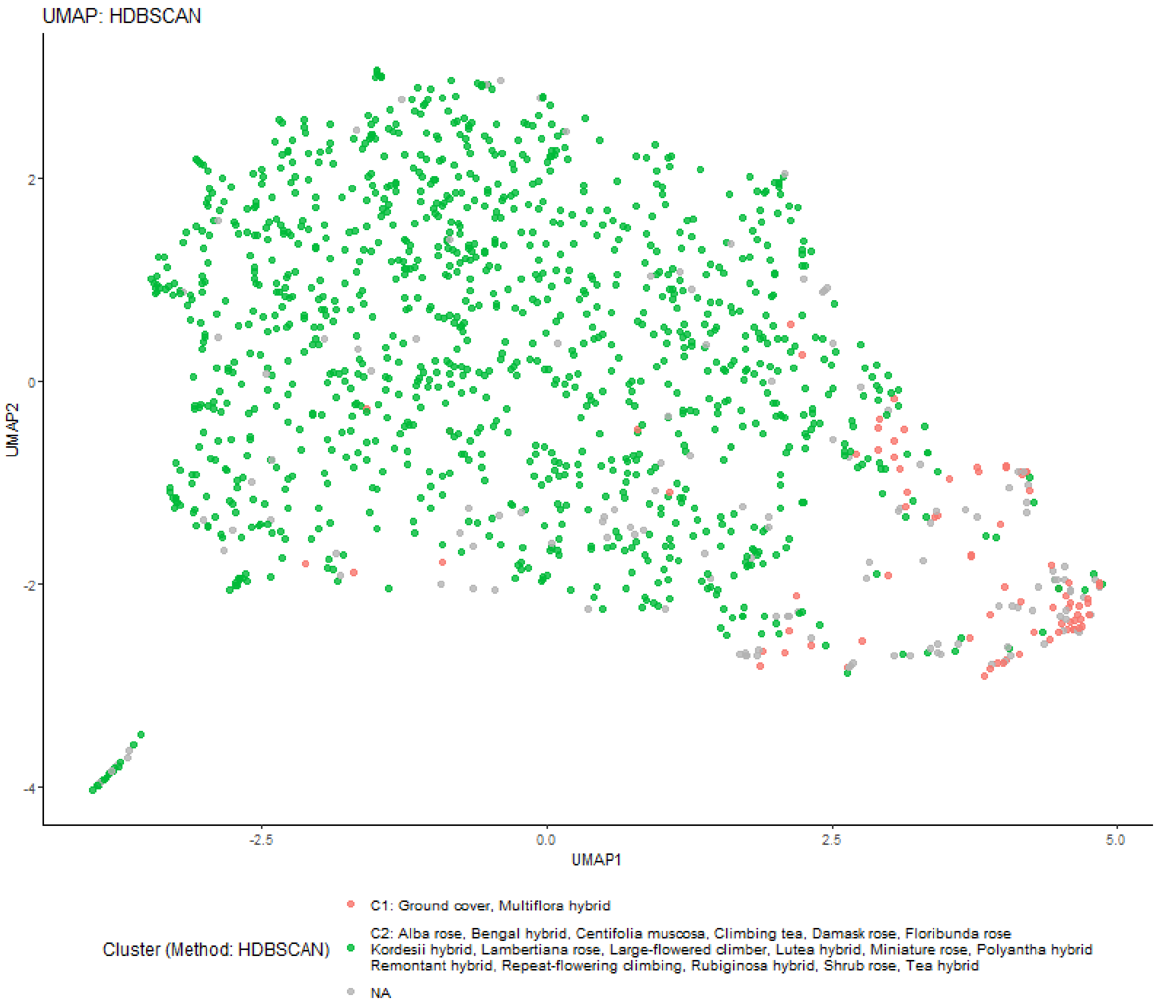

UMAP: HDBSCAN
UMAP2
2
0
-2
-4
-2.5
0.0
2.5
5.0
UMAP1
Cluster (Method: HDBSCAN)
C1: Ground cover, Multiflora hybrid
C2: Alba rose, Bengal hybrid, Centifolia muscosa, Climbing tea, Damask rose, Floribunda rose Kordesii hybrid, Lambertiana rose, Large-flowered climber, Lutea hybrid, Miniature rose, Polyantha hybrid Remontant hybrid, Repeat-flowering climbing, Rubiginosa hybrid, Shrub rose, Tea hybrid
NA

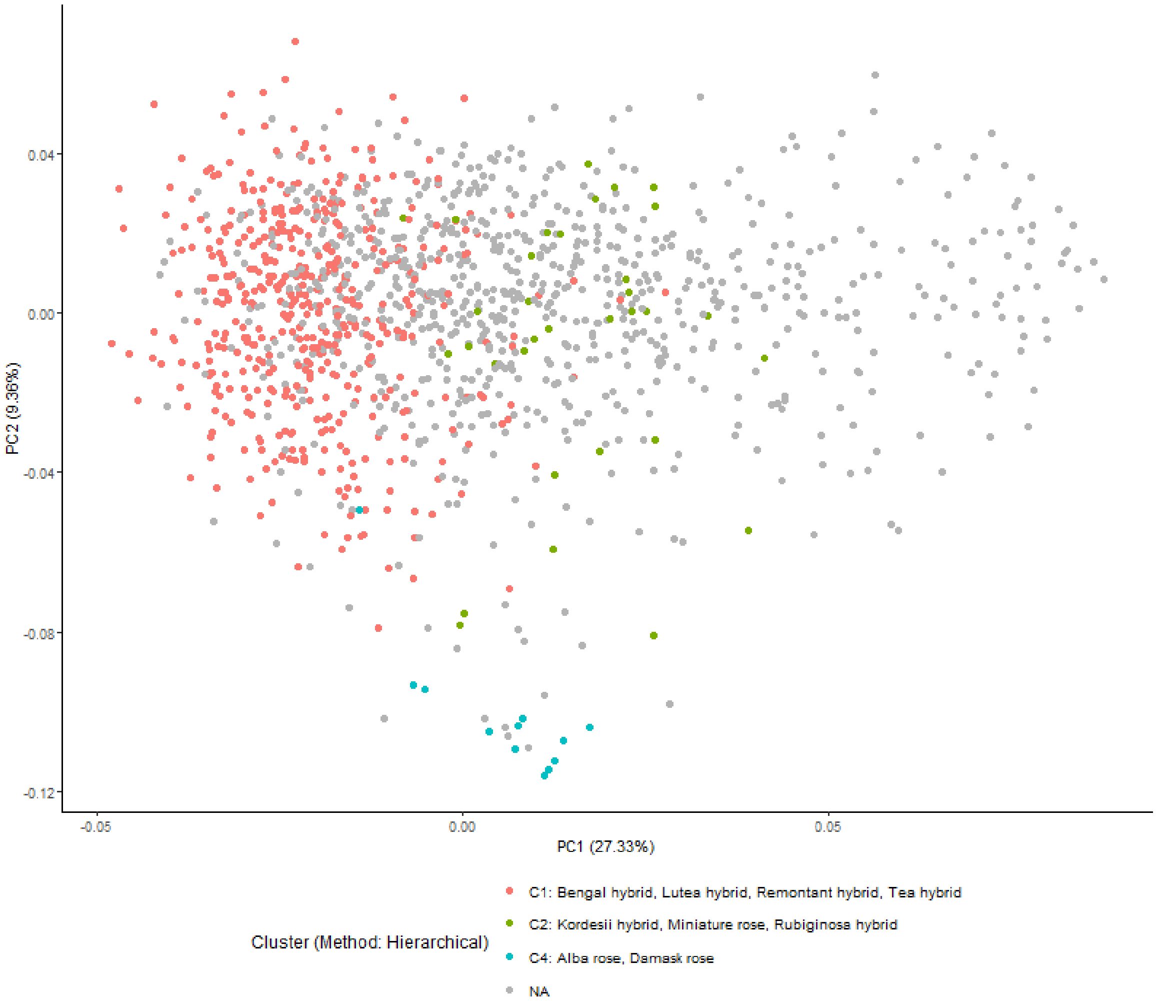

PC2 (9.36%)
0.04
0.00
-0.04
-0.08
-0.12
-0.05
0.00
0.05
PC1 (27.33%)
Cluster (Method: Hierarchical)
C1: Bengal hybrid, Lutea hybrid, Remontant hybrid, Tea hybrid
C2: Kordesii hybrid, Miniature rose, Rubiginosa hybrid
C4: Alba rose, Damask rose
NA

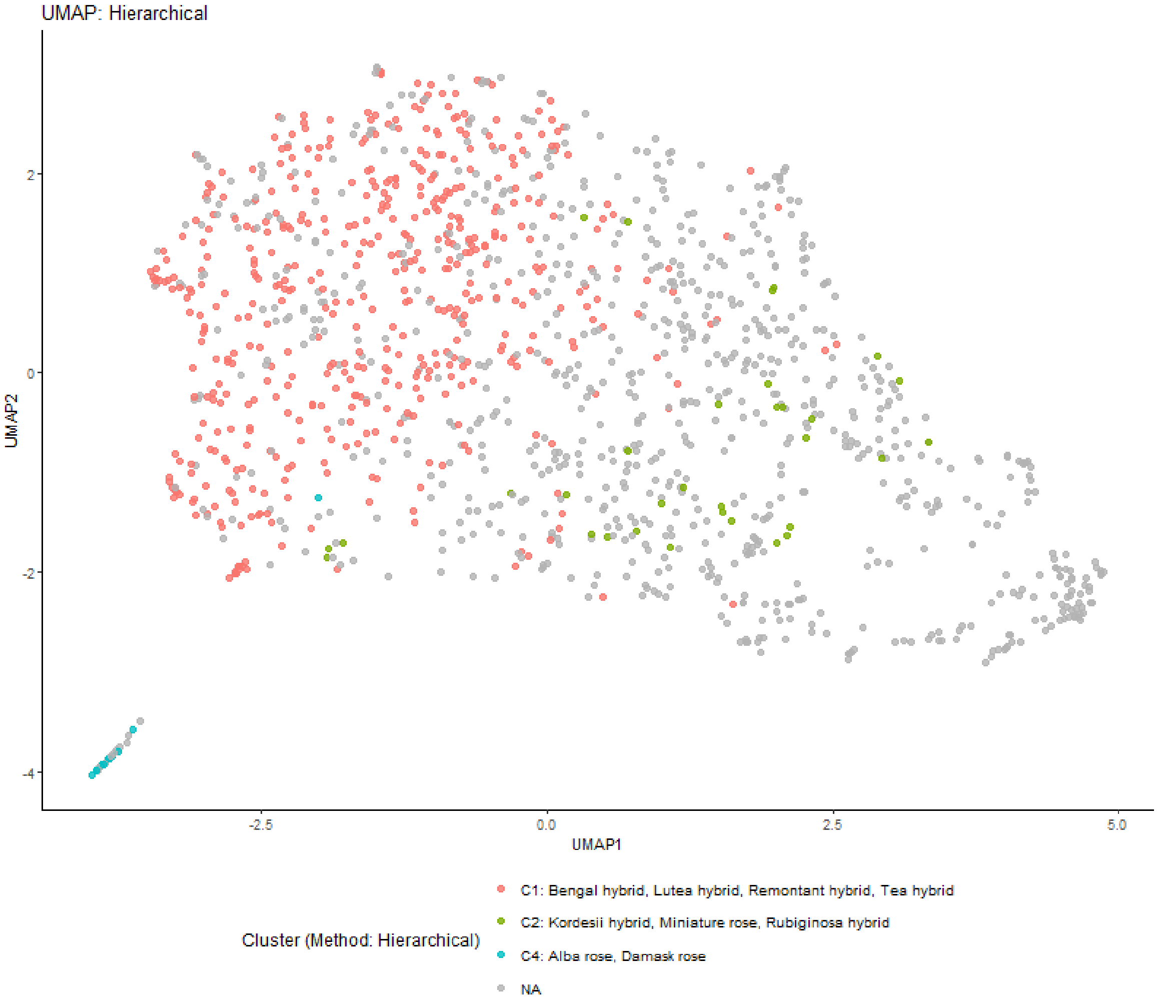

UMAP: Hierarchical
UMAP2
2
0
-2
-4
-2.5
0.0
2.5
5.0
UMAP1
Cluster (Method: Hierarchical)
C1: Bengal hybrid, Lutea hybrid, Remontant hybrid, Tea hybrid
C2: Kordesii hybrid, Miniature rose, Rubiginosa hybrid
C4: Alba rose, Damask rose
NA

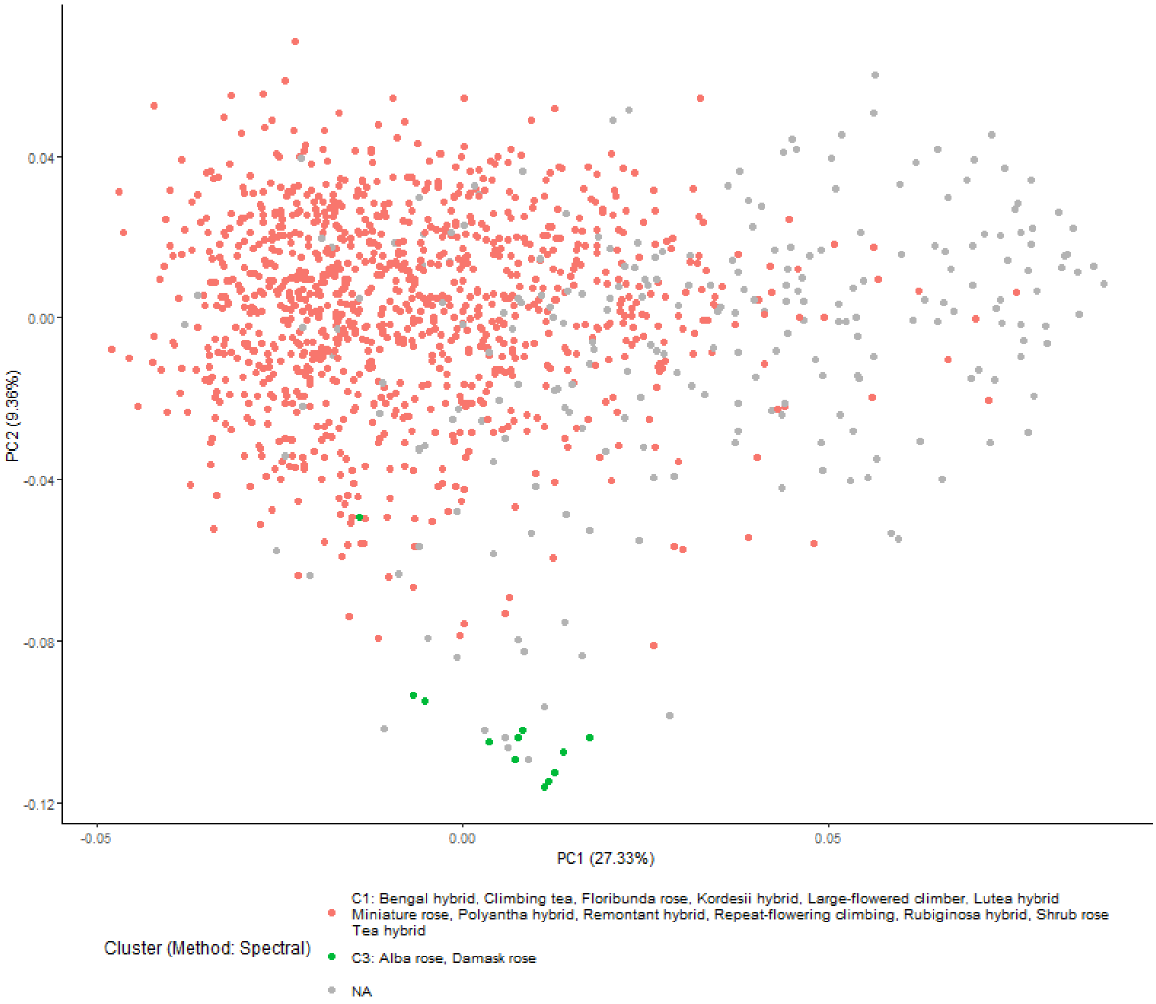

PC2 (9.36%)
0.04
0.00
-0.04
-0.08
-0.12
-0.05
0.00
0.05
PC1 (27.33%)
Cluster (Method: Spectral)
C1: Bengal hybrid, Climbing tea, Floribunda rose, Kordesii hybrid, Large-flowered climber, Lutea hybrid Miniature rose, Polyantha hybrid, Remontant hybrid, Repeat-flowering climbing, Rubiginosa hybrid, Shrub rose Tea hybrid
C3: Alba rose, Damask rose
NA

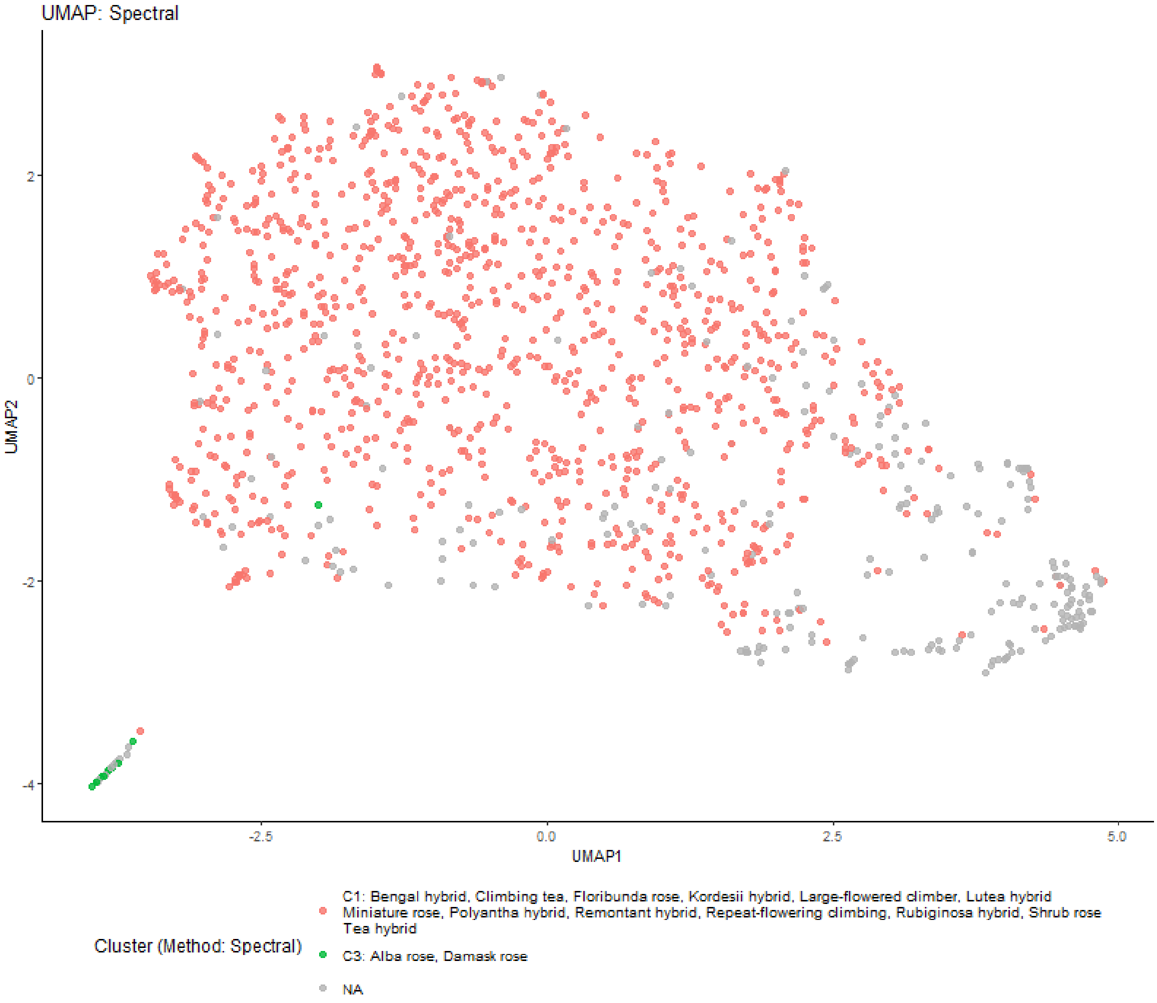

UMAP: Spectral
UMAP2
2
0
-2
-4
-2.5
0.0
2.5
5.0
UMAP1
Cluster (Method: Spectral)
C1: Bengal hybrid, Climbing tea, Floribunda rose, Kordesii hybrid, Large-flowered climber, Lutea hybrid Miniature rose, Polyantha hybrid, Remontant hybrid, Repeat-flowering climbing, Rubiginosa hybrid, Shrub rose Tea hybrid
C3: Alba rose, Damask rose
NA
