# Supplementary material for: Machine learning-based classification of roses using 18 SNP markers for optimized genebank management
Source: Plant Methods. 2026 Jan 6;22:8. doi: 10.1186/s13007-025-01496-0 (PMC12849561; doi:10.1186/s13007-025-01496-0)
Supplement: Supplementary file 6 — Supplementary Material 6. Performance metrics of supervised learning models for rose classes trained on SNP data using original horticultural labels and labels derived on expert knowledge. [file 13007_2025_1496_MOESM6_ESM.pdf]

Additional file for:

Machine Learning-Based Classification of Roses Using 18 SNP Markers for Optimized Genebank Management

Laurine Patzer, Marcus Linde, Thomas Debener

Table 1. **Performance metrics of supervised learning models for rose classes trained on SNP data using original horticultural labels.** For each model, the overall accuracy, balanced accuracy, and Cohen's kappa coefficient are reported alongside the corresponding training set metrics.

Method	Accuracy	Balanced_Accuracy	Kappa	Accuracy_Train	Kappa_Train
svmRadial	0.26	0.53	0.09	0.26	0.11
rpart	0.47	0.53	0.28	0.44	0.24
naive_bayes	0.47	0.58	0.27	0.46	0.27
xgbTree	0.51	0.59	0.36	0.52	0.38

Table 2. Performance metrics of supervised learning models for rose classes trained on SNP data using horticultural labels groups based on expert knowledge. *For each model, the overall accuracy, balanced accuracy, and Cohen's kappa coefficient are reported alongside the corresponding training set metrics.*

Label	Method	Accuracy	Balanced_Accuracy	Kappa	Accuracy_Train	Kappa_Train
Label 1	svmRadial	0.78	0.72	0.61	0.75	0.56
	rpart	0.69	0.62	0.44	0.68	0.43
	naive_bayes	0.73	0.76	0.55	0.70	0.51
	xgbTree	0.82	0.79	0.68	0.77	0.61
Label 2	svmRadial	0.56	0.53	0.08	0.59	0.13
	rpart	0.64	0.57	0.21	0.58	0.11
	naive_bayes	0.64	0.56	0.20	0.65	0.25
	xgbTree	0.67	0.61	0.32	0.61	0.21
Label 3	svmRadial	0.78	0.70	0.22	0.56	0.12
	rpart	0.91	0.66	0.53	0.89	0.43
	naive_bayes	0.91	0.73	0.56	0.91	0.52
	xgbTree	0.90	0.73	0.49	0.91	0.50
Label 4	svmRadial	0.33	0.54	-0.02	0.51	0.04
	rpart	0.73	0.50	0.00	0.74	0.16
	naive_bayes	0.77	0.60	0.22	0.74	0.15
	xgbTree	0.76	0.60	0.28	0.75	0.27

Table 3 Original Labelling of the samples provided by the Europa Rosarium Sangerhausen and different classification approaches based on expert knowledge. *These labels were the basis for Table 2.*

Label ERS	Freq	Label 1	Label 2	Label 3	Label 4
**Acicularis Hybrids**	1				
**Alba rose**	7	old european rose		Gallicanae	Old rose varieties
**Alba rose; Gallicanae**	1	old european rose		Gallicanae	Old rose varieties
**Arvensis hybrids**	1			Synstylae	
**Bengal hybrids**	5	Tea hybrids		Indicae	Chinese and bengal roses
**Bengal roses**	1	Tea hybrids		Indicae	Chinese and bengal roses
**Ground cover roses**	1		Shrub rose		Ground cover and shrub roses
**Ground cover roses to small shrub roses**	23		Shrub rose		Ground cover and shrub roses
**Bourbon hybrids**	1				
**Centifolia muscosa**	10	old european rose		Gallicanae	Old rose varieties
**Centifolia muscosa; Gallicanae**	1	old european rose		Gallicanae	Old rose varieties
**Chinensis**	1			Indicae	Chinese and bengal roses
**Damask rose**	4	old european rose		Gallicanae	Old rose varieties
**Damask rose; Gallicanae**	1	old european rose		Gallicanae	Old rose varieties
**Filipes hybrids**	1				
**Floribunda rose**	281	Polyantha/Floribunda/Multiflora		Modern roses	Modern hybrids
**Gallica**	1	old european rose		Gallicanae	Old rose varieties
**Gallica hybrids**	1	old european rose		Gallicanae	Old rose varieties
**large-flowered climbing rose**	11		Climbing rose	Modern roses	Climbing roses
**Miniature shrub rose**	3		Shrub rose		Ground cover and shrub roses
**climbing remontant hybrid**	1	Tea hybrids	Climbing rose	Modern roses	Climbing roses
**climbing tea hybrid**	11	Tea hybrids	Climbing rose	Modern roses	Modern hybrids
**Climbing rose**	13		Climbing rose	Modern roses	Climbing roses
**Kordesii hybrid**	16	Wichuariana hybrids		Modern roses	Modern hybrids
**Lambertiana rose**	16		Climbing rose		Climbing roses
**Lutea hybrid**	14			Pimpinellifoliae	Wild rose relatives
**Macrantha hybrid**	2			Modern roses	Modern hybrids
**Miniature rose**	12		Miniature rose	Modern roses	Modern hybrids
**Moschata hybrid**	23	old european rose		Synstylae	Wild rose relatives
**Multiflora hybrid**	57	Polyantha/Floribunda/Multiflora		Synstylae	
**repeat-flowering climbing rose**	25		Climbing rose	Modern roses	Climbing roses
**Persica hybrid**	1			Modern roses	Modern hybrids
**Polyantha hybrid**	80	Polyantha/Floribunda/Multiflora		Modern roses	Modern hybrids
**Polyantha rose**	22	Polyantha/Floribunda/Multiflora		Modern roses	
**Portland rose**	1	old european rose			Old rose varieties
**Remontant hybrid**	30	Tea hybrids		Modern roses	Modern hybrids
**Rubiginosa hybrid**	5			Carolinae	Wild rose relatives
**Rugosa hybrid**	3				Wild rose relatives
**Sempervirens hybrid**	1			Synstylae	Wild rose relatives
**Spinosissima hybride**	1			Pimpinellifoliae	Wild rose relatives
**Shrub to climbing rose**	2				
**Shrub rose**	149		Shrub rose		Ground cover and shrub roses

**Shrub rose; Cinnamomeae**	1		Shrub rose		Ground cover and shrub roses
**Tea hybrid**	447	Tea hybrids		Modern roses	Modern hybrids
**Tea rose**	2	Tea hybrids		Modern roses	Modern hybrids
**Wichuariana hybrid**	42	Wichuariana hybrids	Climbing rose	Modern roses	Modern hybrids
**Centifolia**	3	old european rose			Old rose varieties
**Dwarf rose**	2		Miniature rose	Modern roses	Modern hybrids
**Dwarf shrub rose**	2		Miniature rose		Ground cover and shrub roses
